# Investigation and thermodynamic analysis of hydrogen liquefaction cycles: Energy and exergy study

**DOI:** 10.1016/j.heliyon.2024.e37570

**Published:** 2024-09-11

**Authors:** Mehdi Mahboobtosi, D. D. Ganji, Mofid Gorji, Khashayar Hosseinzadeh

**Affiliations:** a Department of Mechanical Engineering, Babol Noshirvani University of Technology, Babol, Iran; b Department of Mechanical Engineering, University of Mazandaran, Babolsar, Iran

**Keywords:** Hydrogen liquefaction, Aspen HYSYS, ORC, LNG, Dual-pressure condensation organic Rankine cycle

## Abstract

The use of hydrogen as a clean fuel has drawn the attention of many scientists due to the problem of energy and environmental pollution caused by fossil fuels. One of the important requirements for expanding the use of hydrogen is the investigation and thermodynamic analysis of liquefaction cycle; this includes the thermodynamic investigation of different cycles of hydrogen liquefaction in pre-cooling and cryogenic cooling. Thermodynamic analysis comprises an examination of the cycle's energy and exergy, as well as the equipment employed. In this research, three liquefaction cycles with different pre-cooling cycle and cryo-cooling cycle have been evaluated. The use of organic Rankine cycle (ORC) and liquefied natural gas (LNG) has also been applied in the cycles and the arrangement of the equipment. Simulations and analyzes have been done in Aspen HYSYS V12. The results show that in the pre-cooling process of cycles 1, 2, and 3, the amount of useful exergy is 49.87 %, 58.87 %, and 61.21 %, respectively, which means that the third cycle uses the input exergy better. Also, in the pre-cooling process of cycles 1, 2, and 3, the amount of exergy loss is 33.86 %, 26.77 %, and 19.73 %, respectively, which means that the third cycle has less exergy loss in the pre-cooling process. The findings indicate that in each of the three cycles, over 50 % of the input exergy is wasted in the cryo-cooling process. Value of specific energy consumption (SEC) for cycle 1,2, and 3 is equal to 6.605 $kWh/{kg}_{LH_{2}}$ , 6.601 $kWh/{kg}_{LH_{2}}$ and 6.618 $kWh/{kg}_{LH_{2}}$, respectively. The three cycles under examination had COP values of 0.19945, 0.19936, and 0.19884, in that order. Also, the values for EXE cycles 1, 2, and 3 are 45.816 %, 45.883 %, and 45.797 %, respectively. Analyzing the energy and exergy of liquefaction cycles is a good step toward increasing cycle efficiency, identifying weak places, and altering cycles to improve efficiency.

## Nomenclature

**Subscripts**
E: expander
Co: cooler
C: compressor
Ev: evaporator
H: heater
HX: heat exchanger
I: equipment number
in: inlet stream
out: outlet stream
c: cold stream
h: hot stream
net: net power
S: separator
R: reactor
P: pump
V: valve

**Nomenclature**
$\dot{E}$: exergy rate
e: mass exergy
$\dot{I}$: exergy loss rate
h: mass enthalpy
$\dot{m}$: mass flow rate
s: mass entropy
ε: exergy efficiency
p: pressure
T: temperature
$\dot{W}$: work rate
$\dot{Q}$: heat rate

**Acronyms**
COP: coefficient of performance
EXE: exergy efficiency of the system
SEC: specific energy consumption
LNG: liquefied natural gas
J-B: Joule-Brayton
ORC: organic Rankine cycle
DORC: dual-pressure organic Rankine cycle
$LH_{2}$: liquid hydrogen
TPD: tons per day
WF: working fluid
EOS: equation of state
LMTD: logarithmic mean temperature difference
UA: surface area multiplied by the overall heat transfer coefficient
LN_2_: liquid nitrogen
N_2_: nitrogen
MR: mixed refrigerant

## 1 Introduction

Global warming poses a significant danger to humanity's life and growth [1]. The extensive use of fossil fuels since the industrial revolution has greatly accelerated global economic growth and the progress of industrialization globally, yet it has also brought about climate change and air pollution change issues on a global scale. Hydrogen, a clean fuel [2], has gotten a lot of interest as an alternative to conventional fuels since it is the most plentiful element on the planet, its products heat and $H_{2}O$, come from clean combustion [3]. Transporting and storing hydrogen as a fuel presents challenges because of its extremely low density [4]. The best method to increase the density of this fuel and lessen the issues brought on by its low density is to liquefy hydrogen. Therefore, developing the hydrogen liquefaction sector will make it easier to use hydrogen energy and use less fossil fuels, which will help to greatly alleviate human concerns related to energy supply. James Dewar succeeded in liquefying hydrogen for the first time in 1898 [5]. He and his colleagues built the country's first 500–600 cubic foot per hour hydrogen liquefier in 1904 [6]. Since liquid hydrogen can be supplied to the market at ambient pressure, the goal of constructing a hydrogen liquefaction plant is to generate liquid hydrogen at atmospheric pressure. Therefore, hydrogen should be reduced from ambient temperature to normal boiling point (20.23K) and change its phase from gas to liquid. As mentioned, hydrogen gas is converted into a liquid to be transported over long distances and has proper safety and is transported by special low temperature tanks. The hydrogen liquid process is divided into four sections in general [7], which is: compression, pre -cooling, cryogenic cooling and liquefaction. The hydrogen pressure in pre-compression has essentially little influence on hydrogen pre-cooling, but it has a substantial impact on hydrogen cooling curves during the cryo-cooling stage [7]. The effectiveness of the cryogenic expansion procedure and the heat exchangers' maximum pressure limit should be taken into account while choosing the precompression pressure. Hydrogen liquefaction usually requires a feed pressure of 15–30 bar [8]. The expansion of high-pressure gases serves as the foundation for cooling in the hydrogen liquid process, and compressing gas is one of the fundamental needs of the hydrogen liquid process in order to expand and reach low temperatures in other sections of the process, including liquid [9]. Joule-Thomson hydrogen coefficients, unlike most gases, are negative at ambient temperature. This means that by reducing pressure (expansion), the hydrogen temperature will rise. As a result, the Joule-Thomson coefficient for hydrogen should be positive prior to the expansion process. This requires that the temperature of hydrogen gas be below its inversion point. In most situations, this occurs near the secondary refrigerant, such as liquid nitrogen in the pre-cooling portion [7,9,10]. Some of the ways of pre-cooling are as closed nitrogen ring cycle, Brayton helium/hydrogen system and cycle of mixed refrigerant systems. Reducing the temperature of hydrogen from the pre-cooling temperature to the temperature close to the boiling point is done in the cryogenic cooling section. The basis of this cooling is based on two methods. If cooling is based on co-enthalpy expansion in the Joule-Thomson valve, hydrogen liquefaction has been done by Linde-Hampson method, and if cooling is based on co-entropy expansion in the expander, hydrogen liquefaction has been done by Claude method. One of the challenges in hydrogen liquefaction is its high economic cost. Hydrogen liquefaction is economically very expensive and its production requires a lot of energy due to the low efficiency of existing equipment. Different equipment is used in a liquid hydrogen production unit. The efficiency of each of the equipment used is effective in the overall efficiency of the process. In every hydrogen liquefaction process, regardless of the production method, equipment such as compressors, heat exchangers, coolers, Joule-Thomason valves or expanders, pumps and pipelines are used. According to these challenges, modifying hydrogen liquefaction technologies to improve liquefaction cycle efficiency and minimize power consumption per kilogram of liquid hydrogen production would lower liquid hydrogen production prices. In countries with peaking systems and countries that import liquefied natural gas, the use of wasted cooling in the liquefied natural gas evaporation system is possible and very important because the use of this wasted cooling in combination with other cycles increases the efficiency of the cycles and costs are reduced [11]. In several of the early hydrogen liquefiers, the hydrogen was pre-cooled using $LN_{2}$. Pre-cooling was done progressively using liquid air, LNG, $N_{2}$, $C_{3}H_{8}$ (propane), and MR. Against the global energy transition towards decarbonization and zero-carbon targets, hydrogen (H_2_) has emerged as a promising medium for energy storage and transportation in large-scale systems. Simultaneously, liquefied natural gas (LNG), serves as a transitional resource in moving towards cleaner energy sources. Consequently, the integration of LNG into liquid hydrogen (LH_2_) has garnered significant attention in recent years. Zhang et al. [12] comprehensively reviews current research on LH2 production processes by integrating the energy mass of LNG. These processes, which include pre-cooling H_2_ utilizing the cold energy released from LNG regasification, producing blue H_2_ from natural gas (NG) derived from LNG regasification, synergistic liquefaction of NG and H_2_, and simultaneous production of LNG and LH_2_ from industrial by-products, can markedly increase the thermodynamic efficiency. Kuendig et al. [13] provided the first report on the use of LNG in a hydrogen pre-cooling process. According to the findings, a pre-cooling operation using LNG at 135 K could save around 11 percent of the power, but a pre-cooling operation using $LN_{2}$ save 25 percent. Yun [14] investigated the use of wasted cooling in LNG conversion process into gas in the hydrogen liquefaction cycle. Thermodynamic analysis including calculation of efficiency coefficient and minimum specific energy consumption was done for the proposed models. In the studied cycle, 15 tons of liquid natural gas were needed for each ton of liquid hydrogen production. In the first stage, hydrogen was pre-cooled using liquid natural gas recovery cooling, and then at −253 °C, liquid hydrogen was prepared for storage after passing through the separator. Chang et al. [15] examined the hydrogen liquefaction cycle's efficiency with helium cooling cycle. In their study, in the pre-cooling part, liquid natural gas recovery system cooling was used. In the liquefied natural gas recovery system, two working pressures of 100 and 7000 kPa were investigated. The hydrogen liquefaction cycle was more efficient when the operating pressure in the LNG line was lowered. In the pre-cooling plan that Riaz et al. [16] suggested, the LNG was first compressed to 30 MPa and then inflated to 7 MPa after absorption the heat from the hydrogen and recovering a portion of the power. On the other hand, Bian et al.'s [17] straight expansion pre-cooling cycle proved to be more effective. By raising the pressure LNG to around 200 K, more cold energy could be utilized during power recovery, which decreased the need for LNG. Yang et al.'s study [18] investigated three different approaches to hydrogen pre-cooling: a cascade precooler that used both LNG and $LN_{2}$ as single precoolers, and two precoolers that used LNG and $LN_{2}$ as single precoolers. The SEC for liquefaction dropped from 13.58 to 11.05 $kWh/kg_{LH_{2}}$, according to the findings. According to the results, the cascade precooler has a more efficient configuration. A cycle with cascade pre-cooling using LNG and $LN_{2}$ in a hydrogen liquefier was presented by Bi et al. [19]. This liquefier SEC is 7.948 $kWh/kg_{LH_{2}}$, which was 26.3 % less than that of the liquefaction using LNG. Furthermore, LNG was put through a pre-cooling stage using a MR by Cho et al. [20], which led to a decrease in SEC from 4.36 to 4.07 $kWh/kg_{LH_{2}}$. In order to lower the energy used in the whole hydrogen liquefaction process, Ghorbani et al. [21] designed an LNG gasification method for hydrogen pre-cooling. According to the findings, the integrated structure's SEC and COP were 4.772 $kWh/kg_{LH_{2}}$ and 0.175, respectively. 2008 saw the introduction of the J-B cascade quadruple cycle in a liquefaction process [22]. Four J-B cycles have varied helium expansion pressures. According to an analysis of the hydrogen-to-helium heat exchange process, the ideal helium expansion ratios in each of the four J-B cycles rise as the temperature of hydrogen decreases. Cascade J-B cycles with identical expansion ratios are often used in modern hydrogen liquefaction, despite the outstanding efficiency of cascade J-B cycles with varying expansion ratios [23,24]. To decrease heat loss, Asadnia and Mehrpooya [25]. Heat exchangers in six J-B cascade cycles are included in one cycle. SEC and COP of the improved process were 7.69 $kWh/kg_{LH_{2}}$ and 0.1710. LNG's cold energy used not only for direct pre-cooling of hydrogen but also to provide electricity that powers the operations involved in hydrogen liquefaction. Three cycles are used to generate power: the ORC, the Brayton cycle, and the direct expansion cycle [26]. With ORC's assistance, LNG's cold energy usage enhanced in comparison to the straight expansion cycle [27]. To produce power, the WF in ORC is condensed, compressed, gasified, and then expanded in a sequential manner [28]. Yilmaz et al. [29] have planned, investigated and analyzed the production of clean and sustainable electricity and hydrogen with three renewable energy sources, a hybrid power plant with the help of wind-solar-geothermal. The developed cycle of their study includes a solar photovoltaic (PV) unit, A triple flash geothermal power plant, wind turbines for electricity generation, and a hydrogen production and liquefaction unit. The results of their investigation show that the power rate of the developed power plant is 9942 kW. Hydrogen production rate and stability index are calculated as 0.02143 kg/s and 2.003 respectively. Finally, the proposed hybrid cycle includes energy and exergetic performance of 32.755 % and 50.06 %, respectively. Bian et al. [30] proposed a new hydrogen liquefaction process based on solar energy using a solar heat pump system. An Absorption Refrigeration System (ARS) is added to the mixed refrigerant precooler and Joule-Brayton deep cooler to cool the compressor discharge during the hydrogen liquefaction process. In addition, a solar-based heat pump system is proposed to provide stable heat flow to ARS for the first time, and a two-circuit organic Rankine cycle to recover residual heat from solar energy at low temperature and high temperature. The results show that LH_2_ production is 98 tons per day with specific energy consumption (SEC), coefficient of performance (COP) and exergy efficiency of 5.633 kWh/kg_LH2_, 0.217 and 53.15 %. To improve thermodynamic efficiencies, reduce economic costs and CO2 emissions, Chen et al. [31] proposed a new hydrogen liquefaction process coupled with mixed refrigerant (MR) pre-cooling cycle and steam methane reforming (SMR) is proposed. Furthermore, liquefied natural gas (LNG) cold energy is used to precool the process and evaporated natural gas is used as raw material for SMR to produce hydrogen. To improve the cold energy utilization rate of liquefied natural gas (LNG), reduce the cost of hydrogen liquefaction, recover waste heat and reduce carbon dioxide emissions, Qiao et al. [32] designed. The result show that the values of SEC, COP and exergy efficiency were 5.93 kWh/kg_LH2_, 0.2225 and 53.24 %, respectively. Exergy losses of the system are mainly distributed in heat exchange equipment and compressors.

This research has investigated three proposed cycles. Thermodynamic analysis, exergy analysis, SEC, COP and EXE of the proposed cycles have been investigated. The hydrogen is liquefied in the first cycle, which uses a double-pressure Brayton cascade cycle to cool the hydrogen. Refrigeration cycles using helium and LNG as refrigerants are used in the hydrogen liquefaction system to pre-cool and cryocool hydrogen. In the liquefaction process, ortho to para hydrogen has been converted using four reactors. In the second cycle, an ORC serves as a hydrogen pre-cooling cycle while the liquefaction is connected with the LNG gasification process. Moreover, a refrigeration system with a dual-pressure J-B cycle for cooling hydrogen is included. Two J-B cascade cycles are part of the refrigeration system. The third cycle is a liquefaction process that uses enhanced J-B cascade refrigeration cycles and a DORC, which is aided by the regasification of LNG. In this hydrogen cycle, the feed is pre-cooled by liquefied natural gas. It is then refined, cooled and liquefied through Joule-Brayton cascade cycles. Eventually, liquid hydrogen that satisfies the pressure and parahydrogen conditions is produced. The suggested cycles are modeled in Aspen HYSYS V12.1, a process simulator that combines thermodynamics, engineering techniques, and numerical computation. It has an outstanding database and is extensively used in the design of the chemical, energy, and refining sectors. The Aspen HYSYS compares and evaluates the different parameters of the suggested cycles.

The purpose of this research is to analyze the energy and exergy of hydrogen liquefaction cycles, comprehensively evaluate and improve the thermodynamic performance of these systems. Energy analysis focuses on quantifying overall energy consumption and efficiency, identifying areas of significant energy loss. On the other hand, exergy analysis goes deeper and identifies sources and amounts of irreversibilities in the cycle, which directly contribute to diminishing returns. By understanding exergy degradation in each component and process step, researchers and engineers can identify opportunities for design optimization and improvement. Also, pressure relief valves are used in cycles to reduce the pressure of the produced liquid hydrogen. Also, all heat exchangers have been fully analyzed, which provides researchers with very comprehensive information. UA analysis of heat exchangers, T-s diagram is one of the other things done in this research.

## 2 Conversion ortho-to-para hydrogen

A hydrogen atom is made up of a nucleus containing a proton that accounts for the majority of its mass and an electron that orbits the nucleus. In 1912, a strange phenomenon was noticed during low-temperature hydrogen testing. It was found that, while evaluating hydrogen's heat capacity at cryogenic temperatures, the cooling curve did not match the heating curve. In 1917, the theory of the existence of two states, ortho and para, was proposed. In 1929, the existence of these two molecular states was proved using charcoal catalyst. Hydrogen is either ortho or para depending on the nucleus's spin orientation. One atom's spin orientation is opposite that of the other in the para hydrogen molecule. To put it another way, one turns counterclockwise, and the other, clockwise. However, in the ortho hydrogen molecule, both of the atoms' spin orientations are parallel, or clockwise. While para hydrogen forms more readily at lower temperatures and has a lower energy, the ortho hydrogen form is more excited and has greater levels of energy. The number of nuclear spin states (2I+1) ratio be used to calculate the number of states in a hydrogen molecule. I, which equals 1/2, is the nucleus' quantum spin number in this relationship. With α=+1/2 and β=−1/2 for magnetic quantum numbers, respectively, and I=(1/2+1/2)=1 for ortho hydrogen, this molecular form exists in three states. Hydrogen I=(1/2−1/2)=0 in para and as a consequence there is just one state in this kind of molecule. Because of this, there are three times as many ortho states than para states at room temperature. Consequently, this gas, also known as normal hydrogen, has a 75 % ortho and a 25 % para composition at ambient temperature. When ortho-hydrogen is converted to para-hydrogen, heat is released because ortho-hydrogen has a greater energy level than para-hydrogen. The latent heat of hydrogen vaporization is about 0.218 kcal/mol, while the heat arising from the spontaneously converting of ortho to para is around 0.388 kcal/mol. As a result, this causes some hydrogen to be wasted when storing liquid hydrogen. The rate of loss of stored liquid hydrogen at the beginning is around 1 % of the quantity kept in an hour. After a considerable amount of time, however, 69.3 % of the liquid hydrogen will evaporate, leaving around 30 % of the initial quantity of hydrogen in liquid phase in the storage tank. Consequently, in light of the previously discussed concerns, it is essential to reduce evaporation throughout the procedure of transforming ortho to para hydrogen and to preserve liquid hydrogen for an extended period of time. As a result, a portion of the majority of liquefaction procedures are created specifically for the purpose of converting ortho to para hydrogen. Equilibrium hydrogen consists two forms: para-hydrogen and ortho-hydrogen, whose ratio varies with temperature [33,34]. Ortho-hydrogen changes into para-hydrogen as the temperature drops, releasing heat, and para-hydrogen changes into ortho-hydrogen when the temperature rises [35]. Slowly, once the hydrogen becomes liquefied, the transformation from ortho-to para-hydrogen takes place in storage tanks Numerous investigations have demonstrated that the heat generated in the conversion process caused massive amounts of liquid hydrogen to evaporate, severely restricting the production capacity of a hydrogen liquefaction facility [36]. Catalysts are thus required to accelerate a reaction throughout the liquefaction. By including conversion reactors into the liquefaction at the right temperatures, adiabatic conversion achieved. Using the experimentally recorded equilibrium hydrogen data [37], the conversion rate is corrected throughout the simulation by changing the conversion coefficients, as shown by the following formula [17]:

(1) $$Conversion\left(\%\right)=C_{0}+C_{1}T+C_{2}T^{2}$$

In Equation (1), T represents the temperature in terms of K, $C_{0}$, $C_{1}$ and $C_{2}$ represent the conversion coefficients that are determined for the proposed cycles reviewed in separate tables.

## 3 Process simulation

### 3.1 First proposed cycle of hydrogen liquefaction

**Fig. 1:**
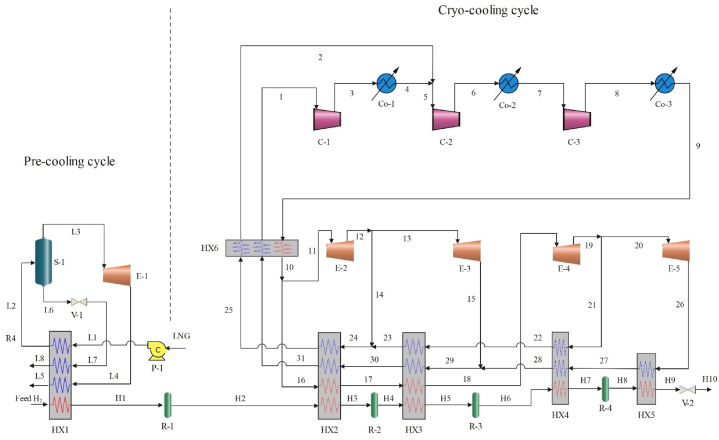
Diagram of the cycle 1 liquefaction cycle.

The first suggested refrigeration cycle uses LNG and helium as refrigerants to complete pre-cooling and cryogenic cooling. Hydrogen cooled via a cryo-cooling process after passing through the pressure relief valve, its pressure drops to 130 kPa. The proposed cycle consists of four ortho-to-parahydrogen conversion reactors, which ensure that the liquid hydrogen produced contains more than 95 % parahydrogen. After passing through the pump, the pressure of LNG reaches 3000 kPa. After flowing through the heat exchanger, in the separator, liquid and vapor phases are separated. Following the reduction of pressure to 120 kPa, liquid phase passes through the HX and its temperature reaches 296.7 K. For the gas phase, the same thing happens in the expander and heat exchanger receives the expander's output. The cooling part of cryo-cooling uses helium as a refrigerant. Helium enters the cooler after passing through the first compressor. These steps are repeated in the second and third compressors. The refrigerant coming out of the last cooler is divided into two parts after passing through the helium self-cooling heat exchanger. Stream 11 enters the second expander and its output is divided into streams 13 and 14. The second expander's mass flow rate at output together with the mass flow rate input to the fifth expander form the total input flow to first compressor. Fig. 1 shows the currents of the first proposed cycle completely. The efficiency and related specifications of the used equipment as well as the conditions of input and output hydrogen and LNG are given in Table 1 [17]. Additionally, Table 2 provides the conversion coefficients for the conversion reactors that are being employed.

**Table 1:** Information about cycle 1.

Parameter	Value	Unit
Equipment condition		
Adiabatic efficiency of pump	75	%
Isentropic efficiency of compressor	80	%
Isentropic efficiency of expander	85	%
Feed hydrogen condition		
Temperature	298.15	K
Pressure	2100	kpa
Para-hydrogen proportion	25	%
Product condition		
Product capacity	1.39	Kg/s
Temperature	21	K
Pressure	130	kpa
Para-hydrogen ratio	>95	%
LNG parameter		
Temperature	113.15	K
Pressure	120	kpa
Components of LNG		Mole %
Nitrogen	0.2	
Methane	91.3	
Ethane	5.4	
Propane	2.1	
i-Butane	0.5	
n-Butane	0.5

**Table 2:** Conversion coefficients of conversion reactors.

coefficient	value	coefficient	value
Reactor 1		Reactor 2	
$C_{0}$	49.5	$C_{0}$	58.25
$C_{1}$	−0.4578	$C_{1}$	−0.4578
$C_{2}$	$1.158\times 10^{-3}$	$C_{2}$	$1.158\times 10^{-3}$
Reactor 3		Reactor 4	
$C_{0}$	68.75	$C_{0}$	101.5
$C_{1}$	−0.4578	$C_{1}$	−0.4578
$C_{2}$	$1.158\times 10^{-3}$	$C_{2}$	$1.158\times 10^{-3}$

### 3.2 Second proposed cycle of hydrogen liquefaction

**Fig. 2:**
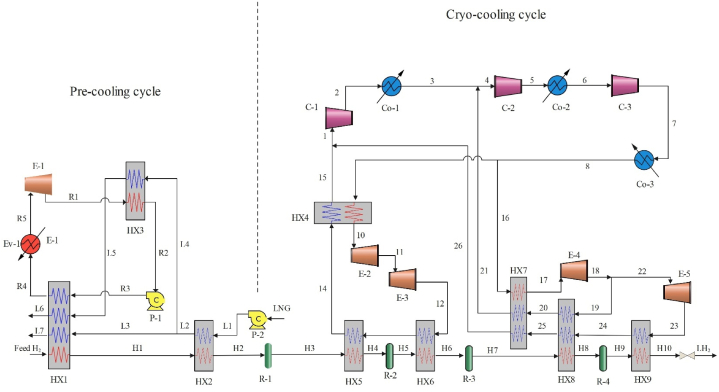
Diagram of the cycle 2 liquefaction cycle.

The proposed liquefaction cycle 2 comprises an LNG pre-cooling cycle as well as enhanced refrigeration processes. LNG is used to pre-cool the hydrogen supply to −156°C. Fig. 2 depicts the diagram of cycle 2 hydrogen liquefaction. With LNG serving as a sink for heat and hydrogen serving as a heat source, the pre-cooling cycle may be thought of as a cryogenic ORC. In order to power the ORC, this system pre-cools hydrogen to −156°C using LNG. First, the LNG pre-cools the hydrogen generated in the HX2 before it is pumped to gasification pressure. The liquid LNG finally separates into two streams during the heat exchange process. The working fluid of HX3 receives cold energy from the LNG (L4) that enters the ORC. Nevertheless, in the HX1 hydrogen cannot be pre-cooled by the condensed working fluid alone. For this reason, some LNG (L3) is required to augment the HX1's cold energy supply. The heat from the hydrogen in the HX1 is subsequently absorbed by the WF and gasified LNG, causing them to heat themselves. To finish gasification, the working fluid of the ORC must next be heated even more in an Ev-1. In the end, the WF will be able to output electricity through an expander 1 to run energy-hungry machinery involved in process. Two cascade J-B cycles are part of the cryogenic refrigeration system. The initial Joule-Brayton cycle involves two expanders (E−2, E−3) linked in series to complete the pre-cooling of hydrogen from −156°C (stream H2) to −227°C (stream H6). The second cycle, known as the double pressure J-B cycle, consists of two expanders (E−4, E−5) that provides two loops capable of independently adjusting the refrigerant's mass flow rate to make heat exchanger composite curves more compatible. Two output flows from two self-cooling helium heat exchangers (HX4-HX7) enter the first compressor (C-1) and then enter the cooler (Co-1). The flow coming out of the cooler is combined with another cold flow coming out of the helium self-cooling heat exchanger (stream 15 and 26) and enters the compressor (C-2, C-3) and subsequent coolers (Co-2, Co-3). For the working fluid in ORC, the combination of R1150 and R290 is used in a ratio of 4:1. The efficiency and related specifications of the used equipment as well as the conditions of input and output hydrogen and LNG are given in Table 3 [38]. The conversion coefficients of the reactors in the proposed cycle 2 are given in Table 4.

**Table 3:** Information about cycle 2.

Parameter	Value	Unit
Equipment condition		
Adiabatic efficiency of pump	75	%
Isentropic efficiency of compressor	80	%
Isentropic efficiency of expander	85	%
Feed hydrogen condition		
Temperature	25	°C
Pressure	2100	kpa
Para-hydrogen proportion	25	%
Product condition		
Product capacity	500	Kg/h
Temperature	−252.2	°C
Pressure	130	kpa
Para-hydrogen ratio	>95	%
LNG parameter		
Temperature	−160	°C
Pressure	120	kpa
Components of LNG		Mole %
Nitrogen	0.2	
Methane	91.3	
Ethane	5.4	
Propane	2.1	
i-Butane	0.5	
n-Butane	0.5

**Table 4:** Conversion coefficients of conversion reactors.

coefficient	value	coefficient	value
Reactor 1		Reactor 2	
$C_{0}$	17.2	$C_{0}$	51.25
$C_{1}$	$-6.082\times 10^{-2}$	$C_{1}$	−0.4
$C_{2}$	$1.036\times 10^{-4}$	$C_{2}$	$1.15\times 10^{-3}$
Reactor 3		Reactor 4	
$C_{0}$	70.6	$C_{0}$	92.7
$C_{1}$	−0.4	$C_{1}$	−0.4
$C_{2}$	$1.15\times 10^{-3}$	$C_{2}$	$1.15\times 10^{-3}$

### 3.3 Third proposed cycle of hydrogen liquefaction

**Fig. 3:**
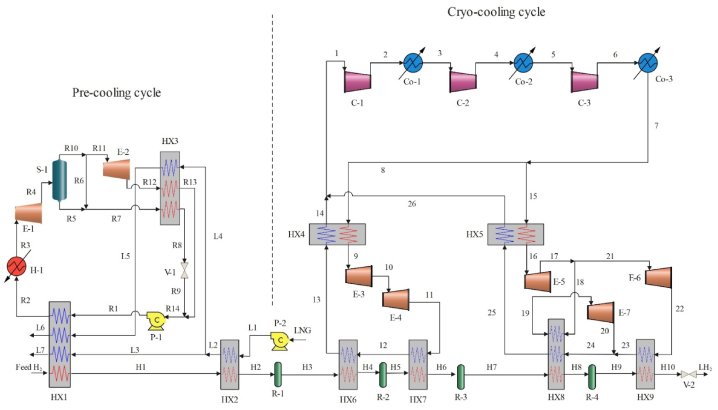
Diagram of the cycle 3 liquefaction cycle.

Fig. 3 displays the third suggested cycle's diagram. The proposed cooling cycle combines DORC, LNG, and helium cascade cooling. Hydrogen is pre-cooled using LNG cold energy, which also powers the DORC to produce electricity. A pump (P-2) brings LNG pressure up to 3000 kPa. It is then used to precool hydrogen, which heats itself (stream L6, L7) to the temperature of regasification (10 °C). HX2 separates the LNG into two streams (L3, L4). Stream L4 serves as the DORC's cold sink, while the other enters HX1 and is utilized immediately to pre-cool hydrogen. Condensed WF enters HX1 in the DORC to pre-cool hydrogen. But in HX1, the working fluid isn't heated at the right temperature, so it goes into H-1 that utilizes seawater. WF expands in E−1 and generates electricity upon regasification A separator for gas and liquid (S-1) is required to separate the two-phase that was formed during the initial expansion. While R5 enters HX3 and is condensed by LNG, R11 expands further in E−2 before entering HX3. In order to concurrently pump two liquid streams via the pump, R8's pressure is lowered via a pressure relief valve and combined with R13's (the pressure of both streams is equal). This cycle considers a dual-pressure J-B refrigeration cycle for the hydrogen cryo-cooling system. Helium is initially compressed in the refrigeration cycle using compressors and coolers in compression systems. It then splits into two streams, which undergo two cascade J-B cycles, namely Streams 8 and 15. Helium is expanded in E−3 and E−4 in the first Joule-Brayton cycle, which cools hydrogen. Following the initial expansion in expander 5, helium in the second Joule-Brayton cycle splits 18 and 21 streams. Stream 18 is extended in expander 7 after hydrogen is liquefied in HX-8, while Stream 21 is used to sub-cool liquid hydrogen just after the second expansion. At the HX8 intake, streams 20 and 23 eventually combine. The enhanced cycle has a further higher helium utilization efficiency than the dual pressure Joule-Brayton cycle. R41 is considered as working fluid in DORC. The efficiency and related specifications of the used equipment as well as the conditions of input and output hydrogen and LNG are given in Table 5 [39]. The conversion coefficients of the reactors in the proposed cycle 3 are given in Table 6.

**Table 5:** Information about cycle 3.

Parameter	Value	Unit
Equipment condition		
Adiabatic efficiency of pump	75	%
Isentropic efficiency of compressor	80	%
Isentropic efficiency of expander	85	%
Feed hydrogen condition		
Temperature	25	°C
Pressure	2100	kpa
Para-hydrogen proportion	25	%
Product condition		
Product capacity	120	TPD
Temperature	−252.2	°C
Pressure	130	kpa
Para-hydrogen ratio	>95	%
LNG parameter		
Temperature	−160	°C
Pressure	120	kpa
Components of LNG		Mole %
Nitrogen	0.2	
Methane	91.3	
Ethane	5.4	
Propane	2.1	
i-Butane	0.5	
n-Butane	0.5

**Table 6:** Conversion coefficients of conversion reactors.

coefficient	value	coefficient	value
Reactor 1		Reactor 2	
$C_{0}$	11.5	$C_{0}$	27.1
$C_{1}$	–	$C_{1}$	–
$C_{2}$	–	$C_{2}$	–
Reactor 3		Reactor 4	
$C_{0}$	53.75	$C_{0}$	80
$C_{1}$	–	$C_{1}$	–
$C_{2}$	–	$C_{2}$	–

### 3.4 Simulation assumptions

The following assumptions are established in order to simulate and assess the proposed process effectively.

• The pressure drop of all system components is ignored, with the exception of pumps, compressors, and expanders.
• Potential and kinetic energy are disregarded, and all processes are in a steady state.
• Each stage of a helium compressor has an identical compression ratio, which can be computed using the first stage's intake pressure and the last stage's exit pressure.
• Exergy losses from mixers and separators are overlooked.
• There is a 100 % rate of conversion between mechanical and electrical energy.

### 3.5 General description

One of the most crucial aspects in hydrogen liquefaction cycles is the cost. Expanders, used to reduce the pressure of the produced liquid hydrogen, are complex devices requiring more maintenance and have a higher initial cost. Therefore, pressure-reducing valves were utilized in this research. Energy and exergy analyses have been conducted separately for the pre-cooling and cryo-cooling process of the cycles to provide more detailed insights for improving liquefaction cycles. The next point is the calculation of UA for heat exchangers. Comparing UA values provides researchers with a very positive perspective for cycle comparison. Additionally, providing tables of conversion coefficients in reactors represents a significant step forward for future researchers, enabling them to accelerate the advancement of this technology.

## 4 Formulation

### 4.1 Energy and exergy

The energy and exergy equations are crucial equilibrium equations for thermodynamic analysis of chemical equipment. The chemical apparatus used as the research object may be classified as an open system under steady-state flow conditions. Thus, the equipment in the cycles, as indicated in Table 7, is suitable for using the exergy and energy calculations. To assess each piece of equipment's performance, exergy efficiency is computed in addition to exergy equations. There are two types of exergy efficiency calculation methods: consumption-production efficiency and input-output efficiency. In the review of cycles, the first case is used. It should be noted that the calculation of exergy efficiency for wasteful equipment is not considered. The formulae used to determine the energy efficiency of various equipment are shown in Table 8.

**Table 7:** Equipment energy and exergy equations.

Equipment	Energy equation	Exergy equation
Compressor	${\dot{W}}_{C}={\dot{m}}_{C}\left(h_{out}-h_{in}\right)$	${\dot{I}}_{C}={\dot{W}}_{C}-{\dot{m}}_{C}\left(e_{out}-e_{in}\right)$
Expander	${\dot{W}}_{E}={\dot{m}}_{E}\left(h_{in}-h_{out}\right)$	${\dot{I}}_{E}={\dot{m}}_{E}\left(e_{in}-e_{out}\right)-{\dot{W}}_{E}$
Pump	${\dot{W}}_{P}={\dot{m}}_{P}\left(h_{out}-h_{in}\right)$	${\dot{I}}_{P}={\dot{W}}_{P}-{\dot{m}}_{P}\left(e_{out}-e_{in}\right)$
Valve	$h_{V,in}=h_{V,out}$	${\dot{I}}_{V}={\dot{m}}_{V}\left(e_{in}-e_{out}\right)$
Heat exchanger	$\sum {\dot{m}}_{h}{\left(h_{\text{in}}‐h_{\text{out}}\right)}_{h}=\sum {\dot{m}}_{c}{\left(h_{\text{out}}‐h_{\text{in}}\right)}_{c}$	${\dot{I}}_{HX}=\sum {\dot{m}}_{HX}\left(e_{in}-e_{out}\right)$
Cooler	${\dot{Q}}_{Co}={\dot{m}}_{Co}\left(h_{in}-h_{out}\right)$	${\dot{I}}_{Co}={\dot{m}}_{Co}\left(e_{in}-e_{out}\right)$
Heater	${\dot{Q}}_{H}={\dot{m}}_{H}\left(h_{out}-h_{in}\right)$	${\dot{I}}_{H}={\dot{m}}_{H}\left(e_{in}-e_{out}\right)$

**Table 8:** Exergy efficiency of equipment.

Equipment	Exergy efficiency
Compressor	$\varepsilon_{C}=\frac{{\dot{m}}_{C}\left(e_{out}-e_{in}\right)}{{\dot{W}}_{C}}$
Expander	$\varepsilon_{E}=\frac{{\dot{W}}_{E}}{{\dot{m}}_{E}\left(e_{in}-e_{out}\right)}$
Pump	$\varepsilon_{P}=\frac{{\dot{m}}_{P}\left(e_{out}-e_{in}\right)}{{\dot{W}}_{P}}$
Heat exchanger	$\varepsilon_{HX}=\frac{\sum {\dot{m}}_{h}{\left(e_{out}-e_{in}\right)}_{h}}{\sum {\dot{m}}_{c}{\left(e_{in}-e_{out}\right)}_{c}}$

### 4.2 SEC, COP and EXE

The energy utilized in the suggested cycles is comprised of both the power supplied by the surrounding environment and the expander's output power. Equation (2) illustrates the total power required for the liquefaction cycles, assuming that the energy-consuming equipment can utilize the expanders' power output without experiencing any loss [39].

(2) $${\dot{W}}_{net}=\sum {\dot{W}}_{C-i}+\sum {\dot{W}}_{P-i}-\sum {\dot{W}}_{E-i}$$

Where ${\dot{W}}_{C}$ represents the power consumed in the compressor, ${\dot{W}}_{p}$ represents the power consumed in the pump, ${\dot{W}}_{E}$ represents the power produced in the expander and ${\dot{W}}_{net}$ represents the net power for hydrogen liquefaction process. SEC is a shorthand the specific energy consumption to liquefy hydrogen. It is a crucial parameter, determined by equation (3), to evaluate the suggested cycle's performance [39].

(3) $$\mathrm{SEC}=\frac{{\dot{W}}_{net}}{{\dot{m}}_{LH_{2}}}$$

Where ${\dot{m}}_{LH_{2}}$ is the mass flow rate of liquid hydrogen and ${\dot{W}}_{net}$ represents the net power for hydrogen liquefaction process. Hydrogen liquefaction may be seen as a process in which energy from the input continues to be transferred to hydrogen via a series of steps until the hydrogen is liquefied. This means that the liquefaction investigated and evaluated in terms of energy. The LNG's entry energy and cold energy provide all of the input energy needed for the suggested process. Some of the energy input is transferred to hydrogen via cooling. A portion of the incoming energy is converted to create power output. The remainder is squandered. As a result, the suggested cycle's exergy equation is as follows [39]:

(4) $$\sum {\dot{W}}_{C}+\sum {\dot{W}}_{p}+\Delta {\dot{E}}_{LNG}=\Delta {\dot{E}}_{H_{2}}+\sum {\dot{W}}_{E}+\sum \dot{I}$$

Where ${\dot{W}}_{C}$ represents the power consumed in the compressor, ${\dot{W}}_{p}$ represents the power consumed in the pump, $\Delta {\dot{E}}_{\text{LNG}}$ is the exergy rate provided by LNG, $\Delta {\dot{E}}_{H_{2}}$ is the exergy rate received by hydrogen, ${\dot{W}}_{E}$ represents the power produced in the expander and $\dot{I}$ is the exergy loss rate of various equipment. The system's COP and EXE used to evaluate the suggested process's thermodynamic efficiency. The efficiency of heat deletion from hydrogen is measured by the COP. The enthalpy difference between the feed hydrogen and the liquid hydrogen is used to determine removed heat during the liquefaction process. Equation (5) shows the COP relation [39].

(5) $$COP=\frac{{\dot{m}}_{LH_{2}}\left(h_{Feed}-h_{LH_{2}}\right)}{{\dot{W}}_{net}}$$

where ${\dot{m}}_{LH_{2}}$ is the mass flow rate of liquid hydrogen, $h_{Feed}$ and $h_{LH_{2}}$ denote the specific enthalpy of feed hydrogen and LH_2_ respectively, and ${\dot{W}}_{net}$ represents the net power for hydrogen liquefaction process.

Equation (6) is used to determine EXE, which is the ratio of the minimum power required to the actual power used. Exergy rate differential between feed hydrogen and liquid hydrogen is calculated to determine the minimum power needed [39].

(6) $$EXE=\frac{{\dot{W}}_{\min}}{{\dot{W}}_{net}}=\frac{{\dot{m}}_{LH_{2}}\left(e_{LH_{2}}-e_{Feed}\right)}{{\dot{W}}_{net}}$$

where ${\dot{m}}_{LH_{2}}$ is the mass flow rate of liquid hydrogen, $e_{LH_{2}}$ and $e_{Feed}$ denote the specific exergy of LH_2_ and feed hydrogen respectively, and ${\dot{W}}_{net}$ represents the net power for hydrogen liquefaction process.

## 5 Result

### 5.1 Results and discussion of the proposed cycle 1

**Fig. 4:**
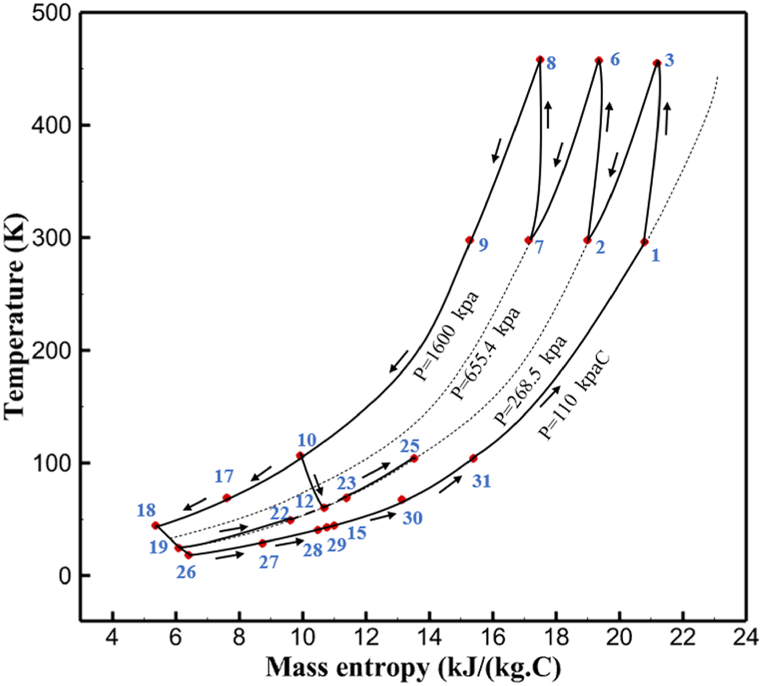
T-s diagram cycle 1.

**Fig. 5:**
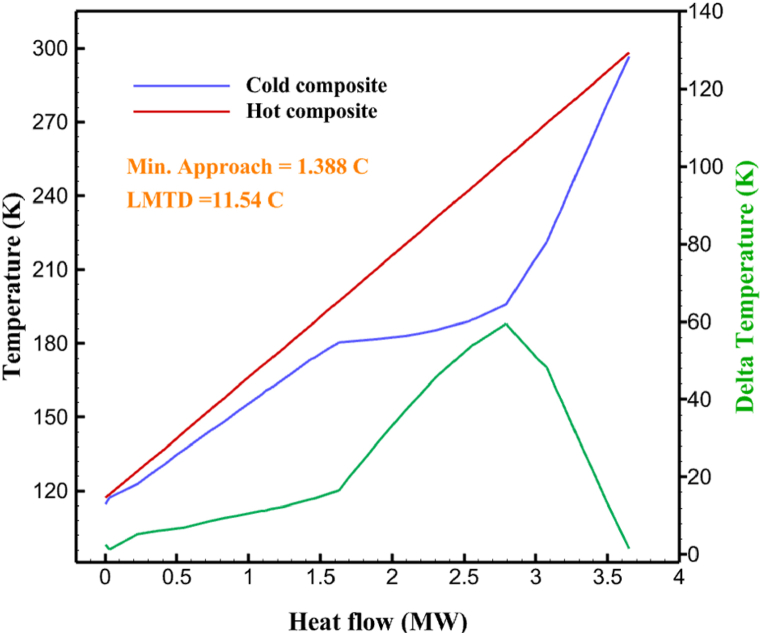

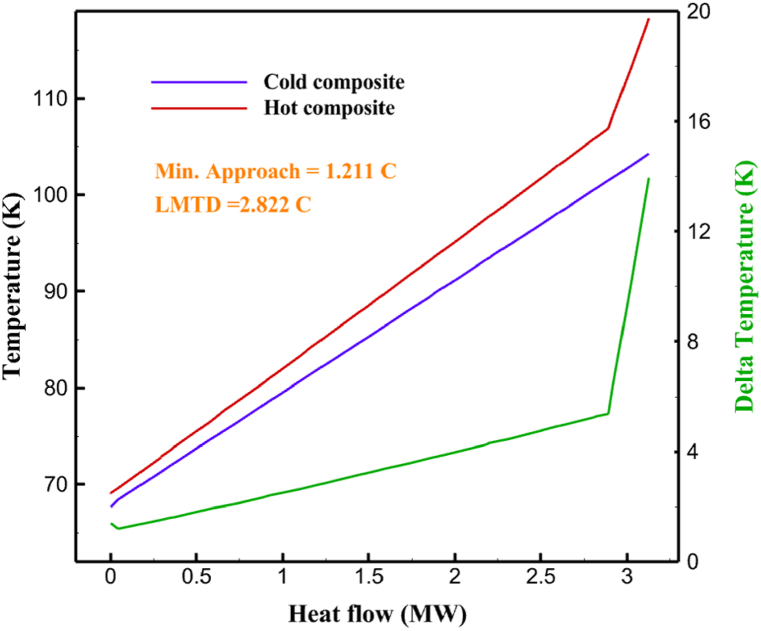

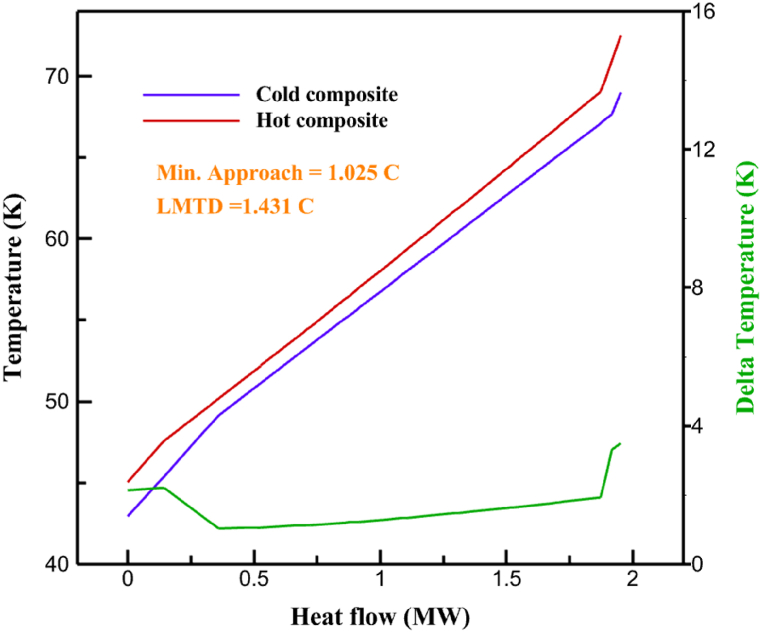

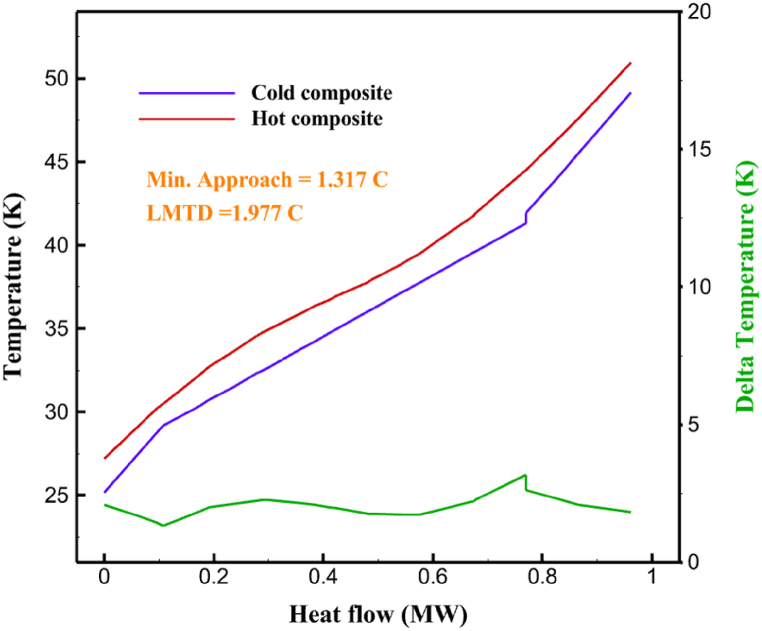

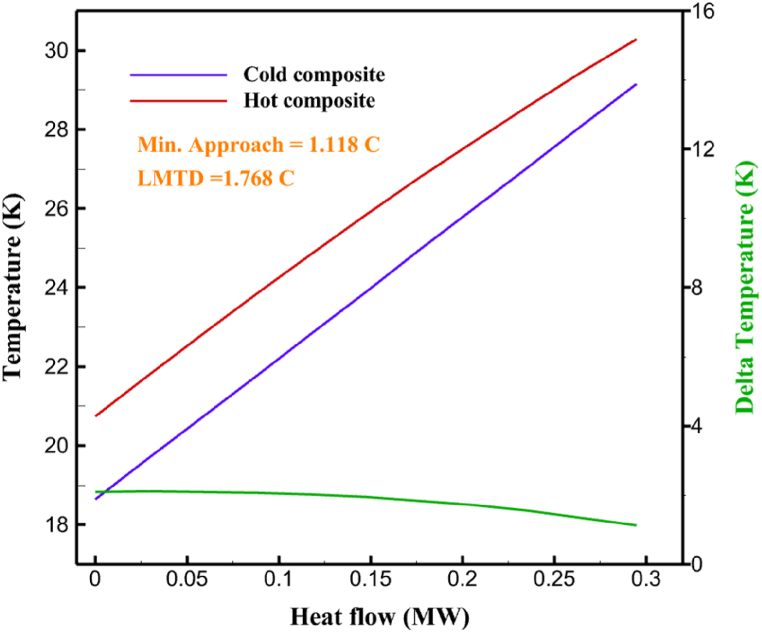

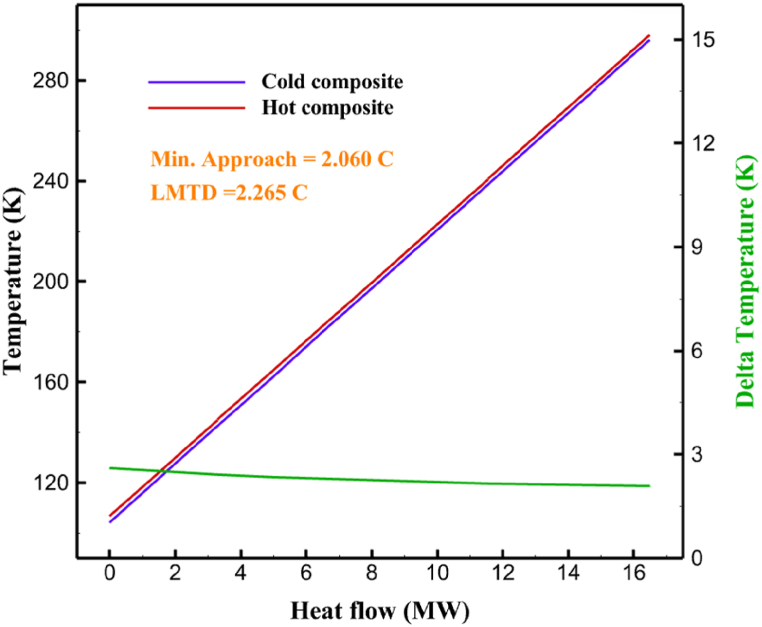
a HX1 information cycle 1. Fig. 5b HX2 information cycle 1. Fig. 5c HX3 information cycle 1. Fig. 5c HX3 information cycle 1. Fig. 5d HX4 information cycle 1. Fig. 5e HX5 information cycle 1. Fig. 5f HX6 information cycle 1.

The thermodynamic analysis of the proposed cycles has been done in Aspen HYSYS. The Peng-Robinson EOS is considered for all three cycles. Table 9 contains all the information required for the thermodynamic analysis of the cycle 1. Table 9 also shows the percentage of parahydrogen in different stages of liquefaction. Finally, the percentage above 95 %, which is one of the existing requirements of the cycle, meets. Fig. 4 shows the temperature-entropy diagram. Four pressure lines are observed in it. It should also be noted that the entropy of the input and output streams in the mixer and separators is the same. Trial and error have been used to determine the cycle's flow parameters. The composite (cold and hot) curves, which are thermodynamic instruments used to gauge the heat exchangers' efficiency in heat transfer, used to determine the reasonableness of the chosen parameters. Fig. 5 a displays the composite cold and hot HX1 curves for hydrogen pre-cooling. The concentrated heat absorption during LNG vaporization makes it difficult to increase the efficiency of HX1, even if the direct LNG expansion cycle reduces LNG usage. The cryo-cooling cycle minimum temperature (Min. Approach) near each heat exchanger (HX2-HX6) is around the limit value (1–2 K), as shown in Fig. 5b to 5 f. The figures show the changes of the cold and hot curves composite with respect to the heat flow. Also, the right axis shows delta temperature. Furthermore, the temperature rise brought on by the conversion of ortho-to para-hydrogen makes it challenging to match the composite (cold-hot) curves at the heat exchanger inlets. The helium self-cooling heat exchanger (HX6) has the largest minimum approach temperature.

**Table 9:** Cycle 1 stream information.

Stream ID	Temperature T (K)	Pressure p (kPa)	Mass flow $\dot{m}$ (kg/s)	Para (%)	Mass enthalpy h (kJ/kg)	Mass entropy s (kJ/kg-K)	Mass exergy e (kJ/kg)
$H_{2}$	298.15	2100	1.39	25	5.84	54.29	3735.79
H1	117.1	2100	1.39	25	−2620.4	40.69	5163.5
H2	118.2	2100	1.39	33.62	−2620.42	42.24	5149.99
H3	69.07	2100	1.39	33.62	−3383.1	33.86	6887.49
H4	72.52	2100	1.39	54.33	−3383.16	37.33	6770.49
H5	47.55	2100	1.39	54.33	−3832.23	29.65	8610.61
H6	50.96	2100	1.39	76.48	−3832.3	32.97	8359.62
H7	27.21	2100	1.39	76.48	−4524.36	14.27	13241.12
H8	30.28	2100	1.39	97.35	−4524.41	17.38	12657
H9	20.73	2100	1.39	97.35	−4736.5	8.983	14947.3
H10	21.02	130	1.39	97.35	−4736.5	10.05	14629.43
LNG	113.15	120	3.67		−5167.14	4.29	957.9
L1	114.7	3000	3.67		−5158.72	4.314	959.01
L2	195.75	3000	3.67		−4644.55	7.424	546.13
L3	195.75	3000	2.931		−4827.65	8.305	557.95
L4	117.11	120	2.931		−4986.56	8.545	327.28
L5	296.75	120	2.931		−4536.18	11.07	25.24
L6	195.75	3000	0.739		−3918.88	3.932	444.02
L7	136.81	120	0.739		−3918.88	4.286	338.57
L8	296.75	120	0.739		−3318.91	7.379	16.35
L9	296.73	120	3.67		−4290.92	10.36	23.45
1	296.09	110	11.1		−10.78	20.8	50.91
2	296.02	268.5	5.44		−11.25	18.95	603.55
3	454.75	268.5	11.1		813.57	21.18	763.21
4	298.15	268.5	11.1		−0.19	18.99	603.51
5	297.45	268.5	16.54		−3.83	18.97	603.51
6	456.84	655.4	16.54		824.43	19.35	1319.71
7	298.15	655.4	16.54		−0.46	17.13	1156.18
8	457.97	1600	16.54		830.11	17.51	1874.65
9	298.15	1600	16.54		−1.06	15.28	1709.02
10	106.87	1600	16.54		−998	9.925	2307.3
11	106.87	1600	6.13		−998	9.925	2307.3
12	60.34	268.5	6.13		−1236.89	10.68	1844.81
13	60.34	268.5	5.75		−1236.89	10.68	1844.81
14	60.34	268.5	0.38		−1236.89	10.68	1844.81
15	44.91	110	5.75		−1316.47	11	1669.22
16	106.87	1600	10.41		−998	9.925	2307.3
17	69.05	1600	10.41		−1196.71	7.629	2793.09
18	45.05	1600	10.41		−1324.4	5.354	3343.61
19	25.14	268.5	10.41		−1421.31	6.083	3029.52
20	25.14	268.5	5.35		−1421.31	6.083	3029.52
21	25.14	268.5	5.06		−1421.31	6.083	3029.52
22	49.15	268.5	5.06		−1295.32	9.604	2105.71
23	69.02	268.5	5.06		−1191.68	11.38	1681.3
24	68.41	268.5	5.44		−1194.84	11.33	1691.81
25	104.26	268.5	5.44		−1008.17	13.52	1224.3
26	18.65	110	5.35		−1453.74	6.399	2902.77
27	29.16	110	5.35		−1398.64	8.744	2258.8
28	40.78	110	5.35		−1338.01	10.49	1797.59
29	42.92	110	11.1		−1326.86	10.76	1729.2
30	67.65	110	11.1		−1198.11	13.13	1151.64
31	104.26	110	11.1		−1007.72	15.38	671.36

**Fig. 6:**
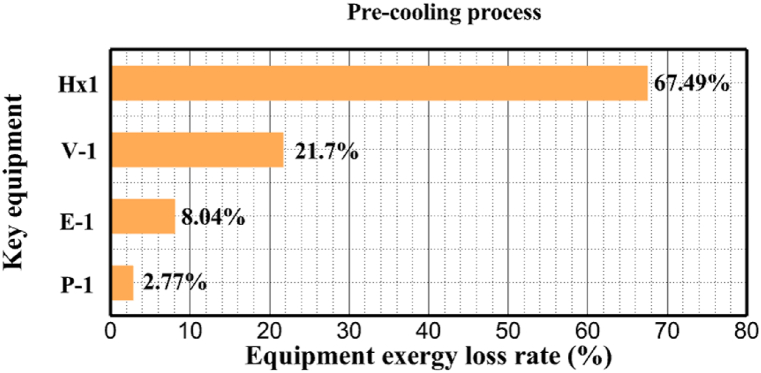

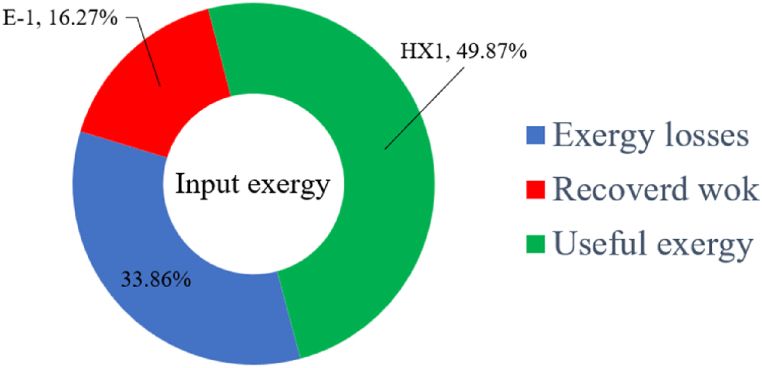

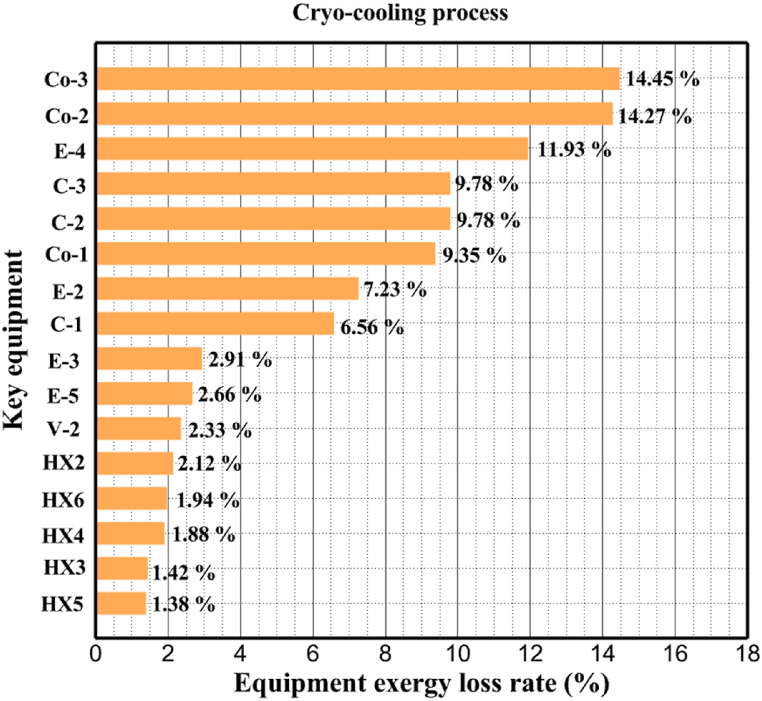

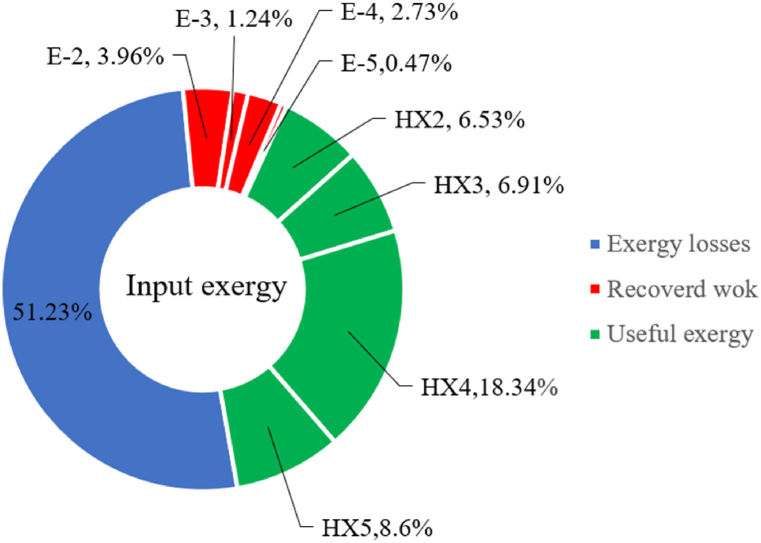

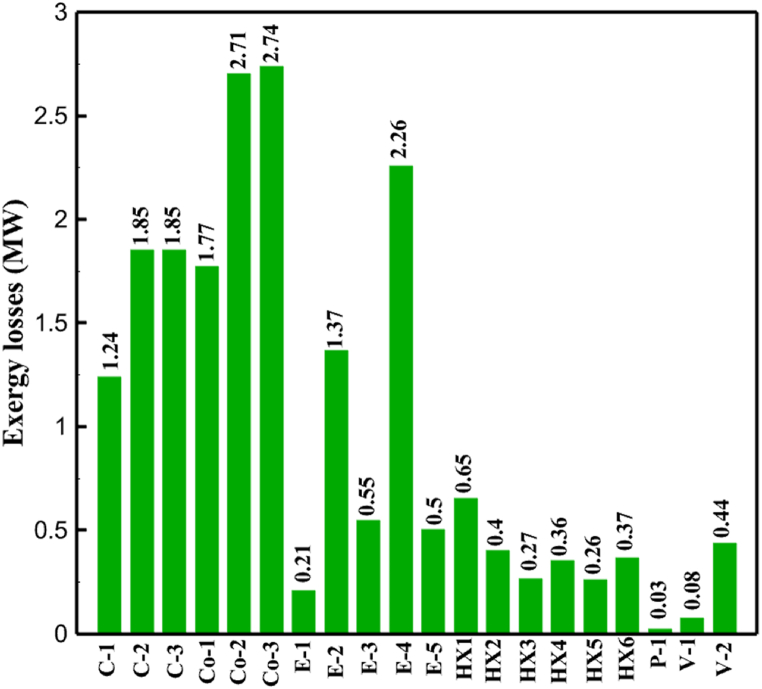

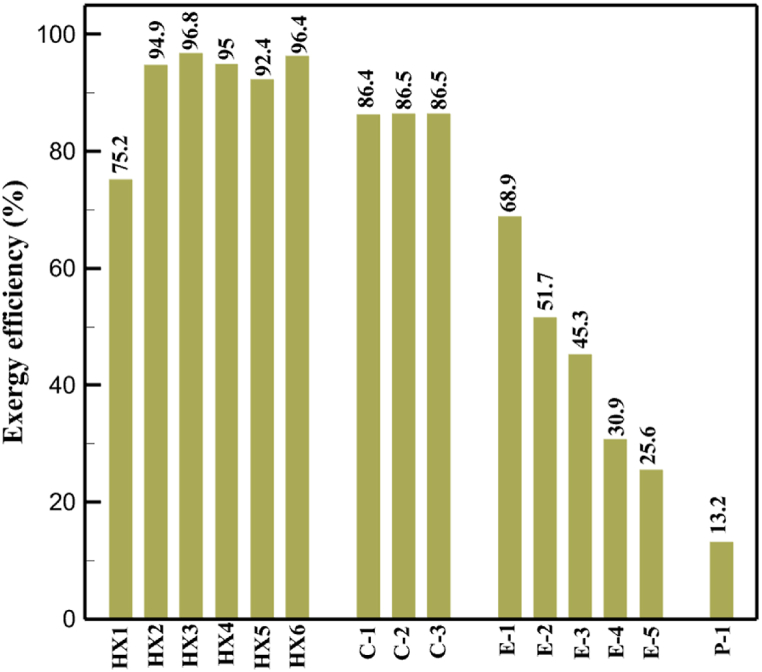
aEquipment exergy loss rate in pre-cooling process cycle 1. Fig. 6b Use of input exergy in pre-cooling process cycle 1. Fig. 6c Equipment exergy loss rate in cryo-cooling process cycle 1. Fig. 6d Use of input exergy in pre-cooling process cycle 1. Fig. 6e Exergy losses each equipment cycle 1. Fog 6.f Exergy efficiency of equipment cycle 1.

Fig. 6a shows the rate of exergy loss rate equipment in the pre-cooling stage. According to the figure, HX1 has the highest loss rate among the equipment. Fig. 6b also shows how the input exergy is used. 48.96 % is useful exergy used by HX1. 33.86 % exergy is losses and 16.27 % exergy is Recovered work by the turbine. In Fig. 6c and 6d, these two issues are examined for the cryo-cooling process. The results show that the highest rate of exergy loss is related to cooler 3. Detailed information about each equipment is also given. Fig. 6d comprehensively shows how the input exergy is consumed. The largest contribution is related to the loses exergy. Also, the utilization percentage of each of the heat exchangers (useful exergy) and the recovery work of each of the expanders are shown in Fig. 6d. Also, Fig. 6e comprehensively shows the amount of exergy losses for all cycle 1 equipment. Cooler 3 (Co-3) with 2.74 MW of exergy losses has the largest share among the equipment. Cooler 2 (Co-2) is in the next rank, which has a large contribution in this cycle with the loss of 2.71 MW. Fig. 6f shows the exergy efficiency of heat exchangers, compressors, expanders and pumps. Heat exchanger 3 (HX3) had the highest exergy efficiency and heat exchanger 1 (HX1) has the lowest efficiency. It should also be noted that compressors have almost equal exergy efficiency. E−1 of the pre-cooling stage has the highest energy efficiency. The calculation power (MW) of the equipment is given in Table 10. According to calculations, compressors have the most power among other equipment. Among the compressors, C-3 has a power of 13.74 MW. Analyzing the exergy and energy of the equipment used in liquefaction allows scientists to reduce the energy consumption per kilogram of liquid hydrogen by modifying and improving the efficiency of cycles and equipment.

**Table 10:** Equipment power cycle 1.

Equipment		Power (MW)
Comps	C-1	9.15
	C-2	13.7
	C-3	13.74
Exps	E−1	0.47
	E−2	1.46
	E−3	0.46
	E−4	1.01
	E−5	0.17
Pumps	P-1	0.03

### 5.2 Results and discussion of the proposed cycle 2

**Fig. 7:**
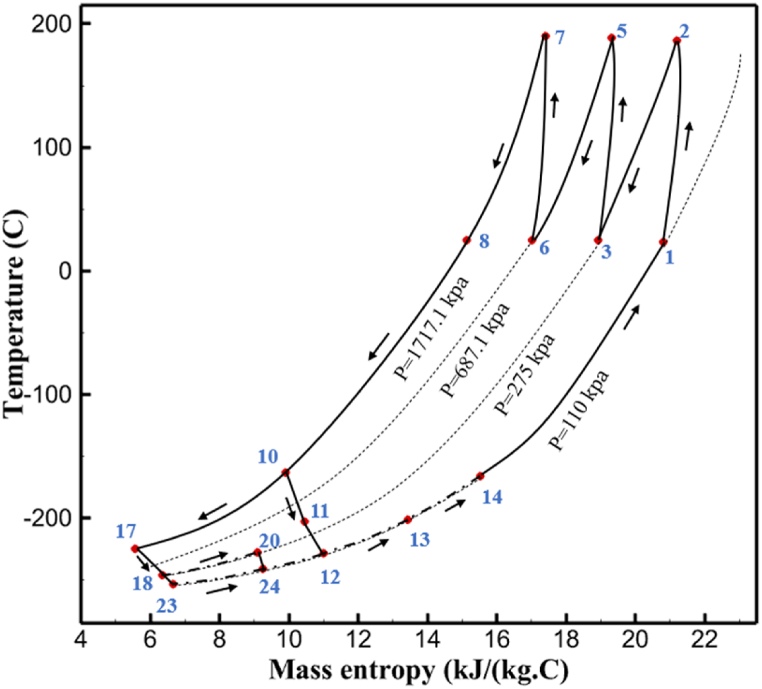
T-s diagram cycle 2.

**Fig. 8:**
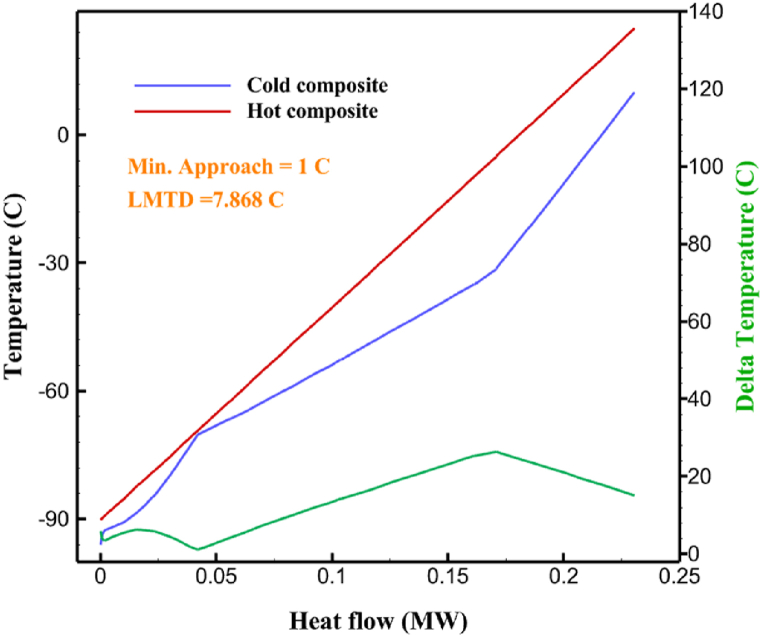

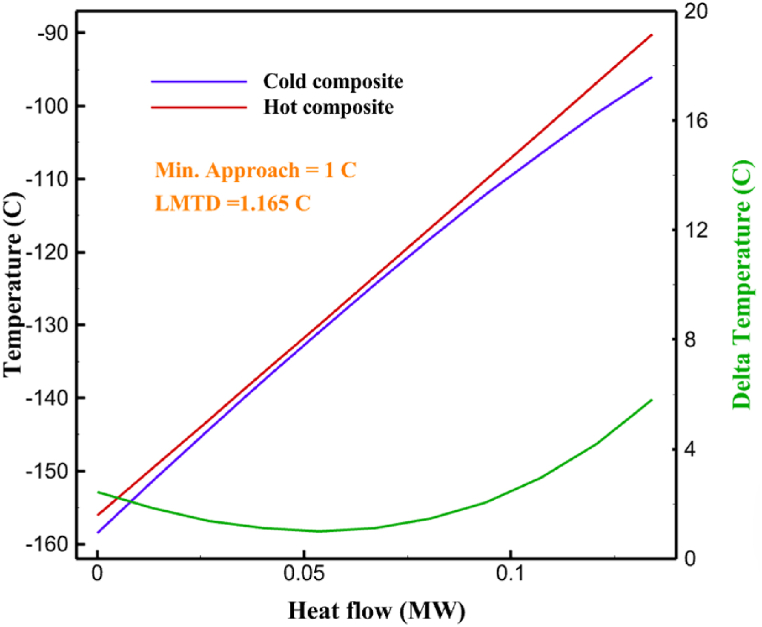

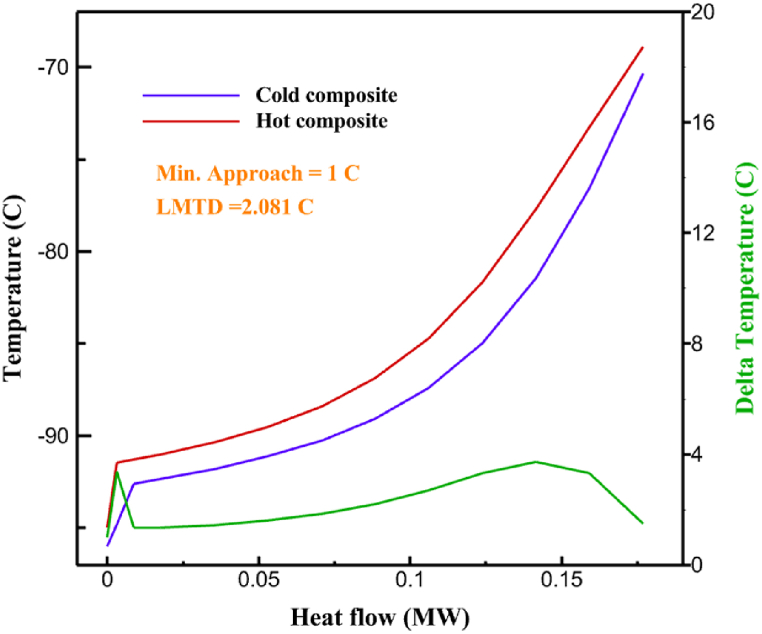

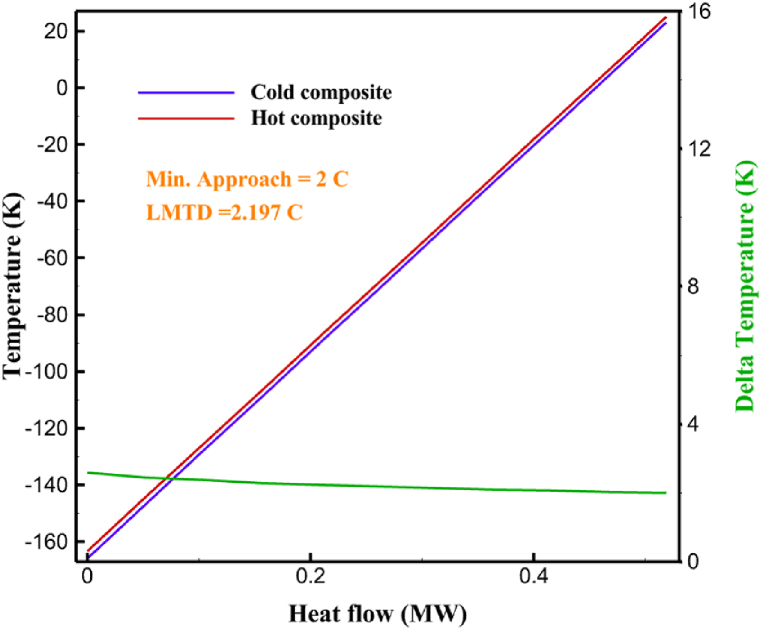

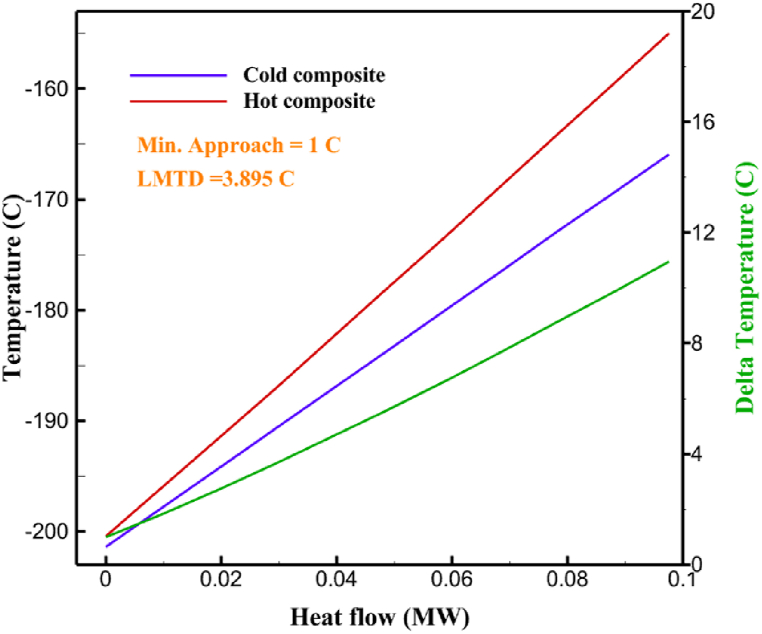

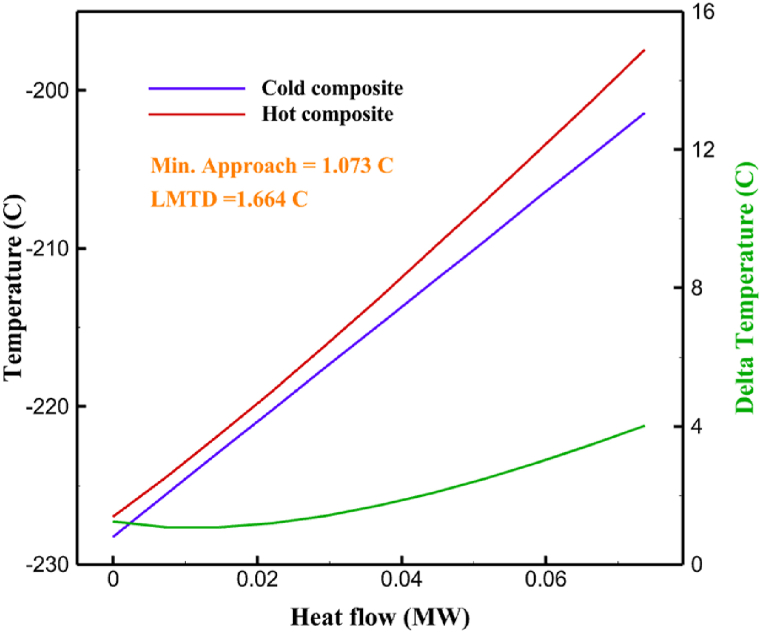

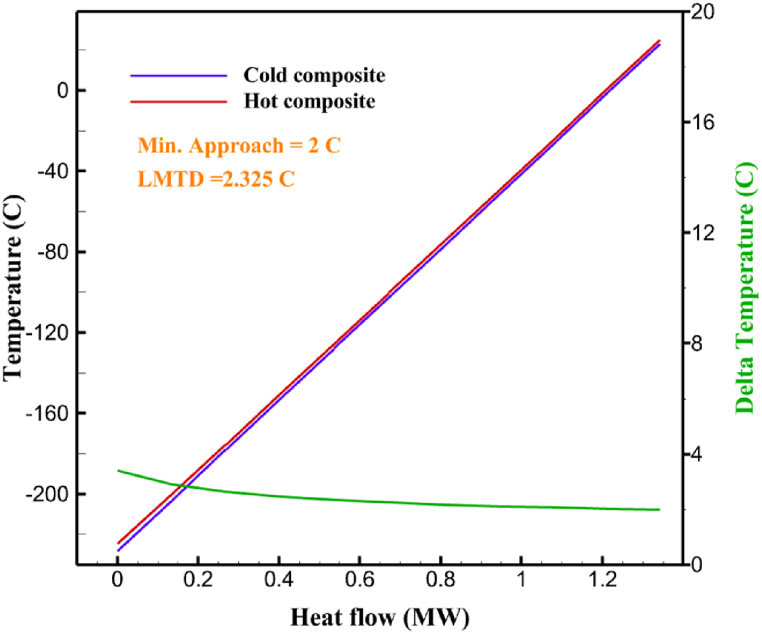

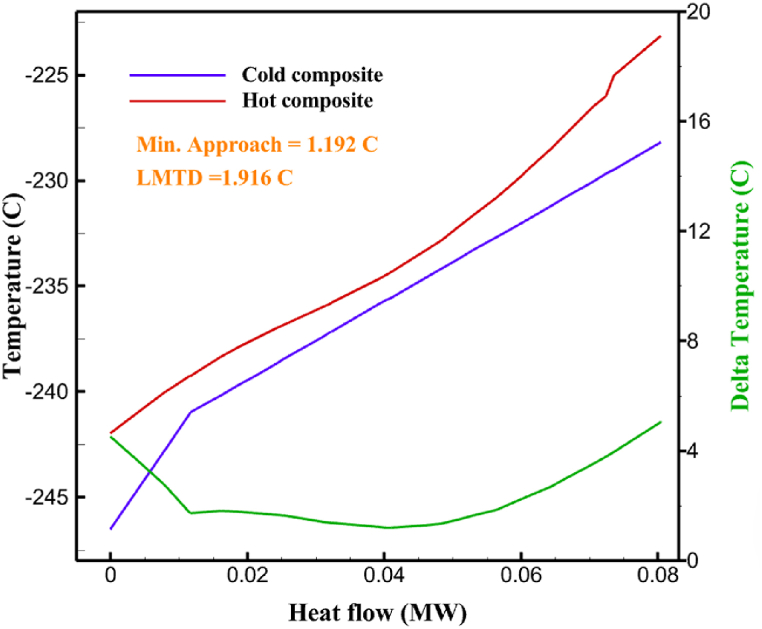

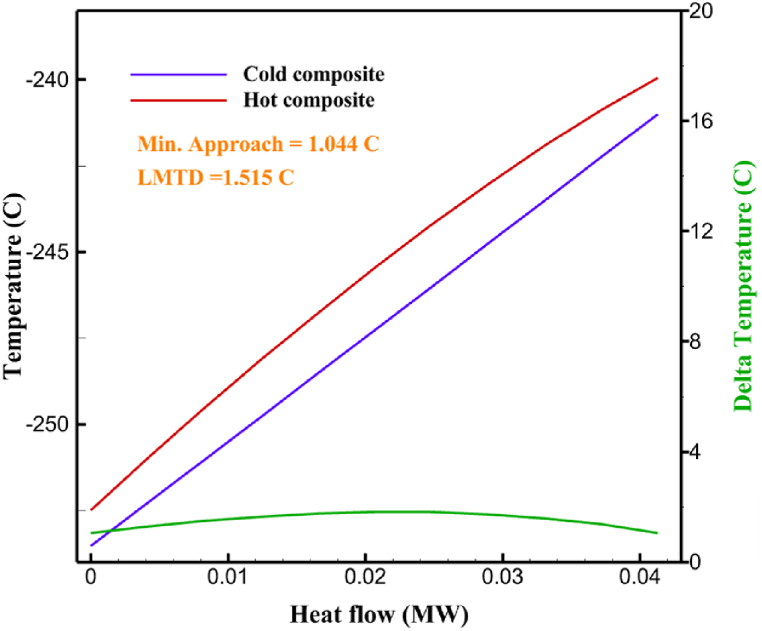
a HX1 information cycle 2. Fig. 8b HX2 information cycle 2. Fig. 8c HX3 information cycle 2. Fig. 8d HX4 information cycle 2. Fig. 8e HX5 information cycle 2. Fig. 8f HX6 information cycle 2. Fig. 8g HX7 information cycle 2 Fig. 8h HX8 information cycle 2. Fig. 8i HX9 information cycle 2.

**Fig. 9:**
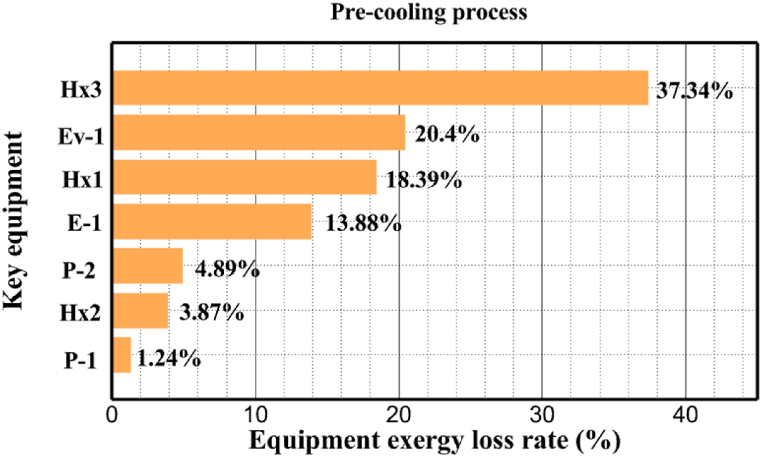

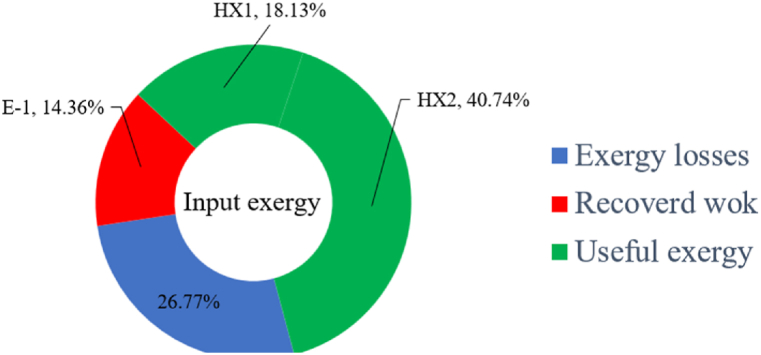

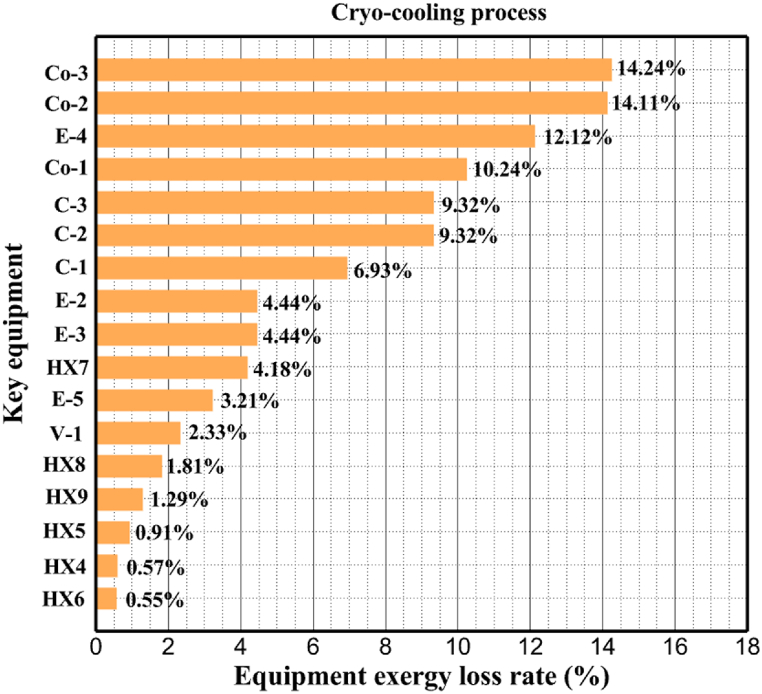

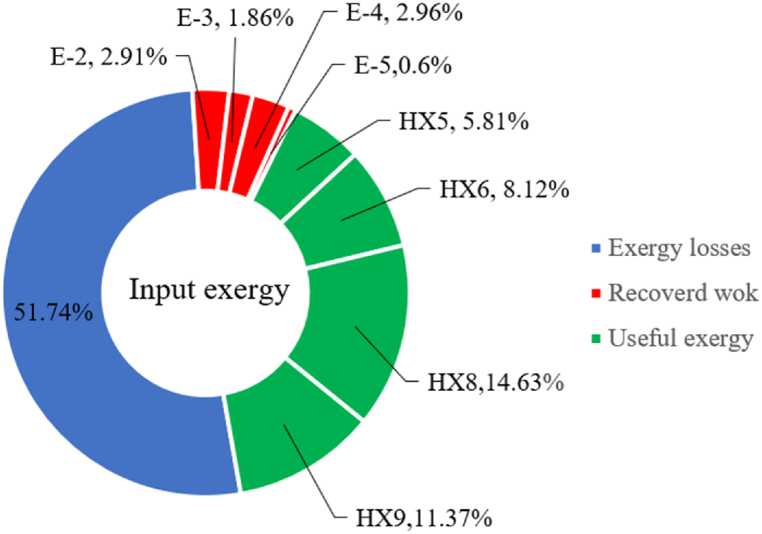

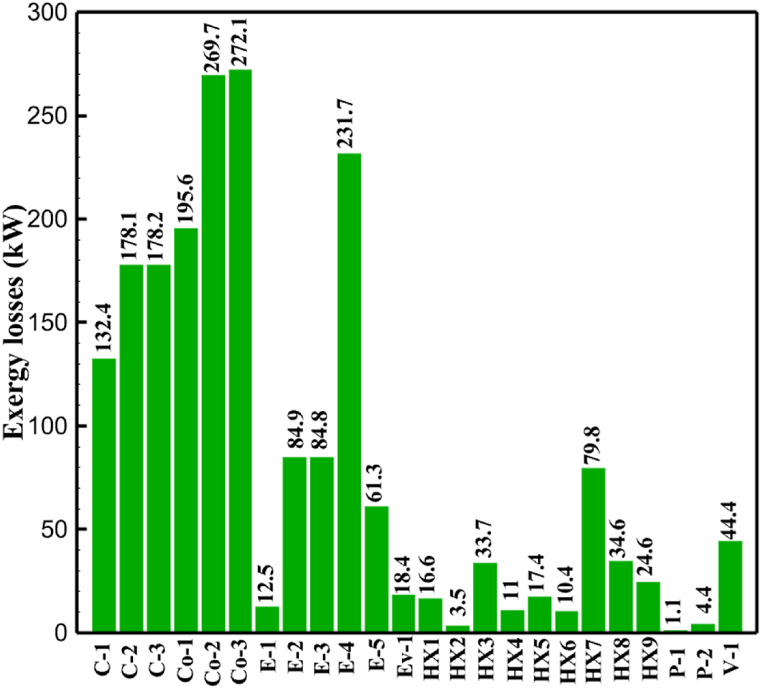

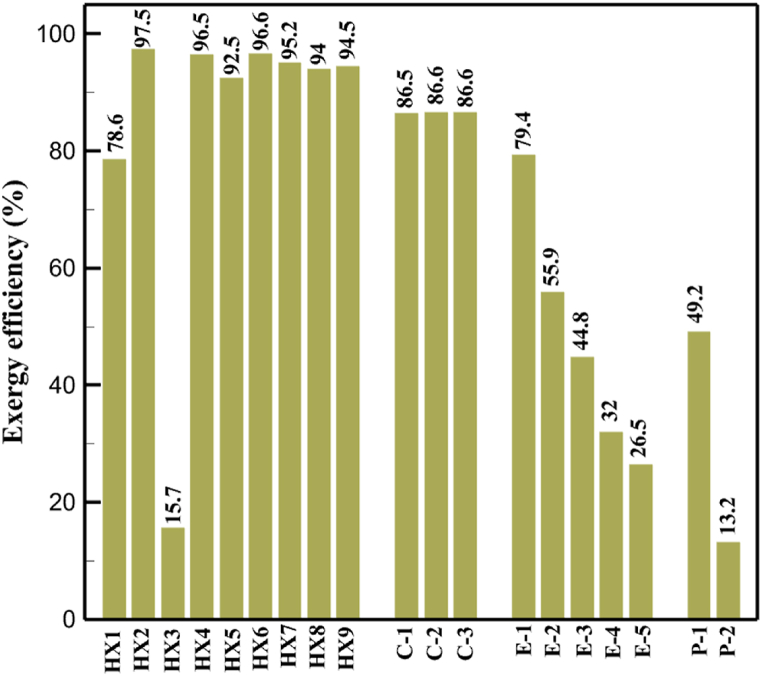
a Equipment exergy loss rate in pre-cooling process cycle 2. Fig. 9b Use of input exergy in pre-cooling process cycle 2. Fig. 9c Equipment exergy loss rate in cryo-cooling process cycle 2. Fig. 9d Use of input exergy in pre-cooling process cycle 2. Fig. 9e Exergy losses each equipment cycle 2. Fog 9.f Exergy efficiency of equipment cycle 2.

Many explanations in the results section of cycle 1 can be expanded for cycles 2 and 3. The information on the cycle's streams is shown in Table 11. Also, the percentage of hydrogen para in different stages of liquefaction is shown. Fig. 7 shows the temperature graph in terms of entropy for cycle 2. What is certain is that four constant pressure lines are visible. As mentioned earlier, the entropy of the incoming and outgoing flows in the mixer and separators are equal. The T-S diagram aids in the visualization of a system's temperature and entropy changes throughout thermodynamic processes, including adiabatic, isentropic, and isothermal processes. This facilitates the comprehension and analysis of these procedures. By giving an accurate and succinct depiction of energy conversions and losses, the T-S diagram also aids in the design and optimization of thermodynamic systems. Fig. 8a to 8i is a diagram that shows the relationship between temperature and heat flow for both cold and hot mixed flows, as well as the temperature difference between these flows for all HXs in the cycle. Shape of the curves (cold and hot composite) shows the heat transfer efficiency between the two streams. The gradual changes in temperature along the axis of the heat flow indicate how effectively heat is transferred from the hot flow to the cold flow. For example, for heat exchanger 1, the LMTD value of 7.868 °C represents the average temperature difference that causes heat transfer, which affects the shape of the temperature profiles. A minimum temperature difference of 1 °C between the hot and cold flow ensures that there is always a driving force for heat transfer, preventing thermal imbalance and maintaining a continuous flow of heat. For other heat exchangers, these results can be easily obtained from the graphs. Fig. 9 a shows the exergy losses of the equipment during the pre-cooling process. Exergy loss indicates system inefficiency, where potential work is wasted. Equipment is rated based on their exergy losses. HX3 has the highest exergy losses (37.34 %). This could be due to the significant temperature difference between the surfaces of the heat exchanger leading to greater irreversibility. P-1 has the lowest exergy losses Fig. 9 b shows that the input exergy is divided into three categories: useful exergy, exergy loss, and recovered work. Most of the useful exergy part is attributed to HX2. This shows that HX2 is very efficient in using the input exergy for useful work or heat transfer. The total exergy loss is 26.77 %. This section shows the total inefficiencies of the system. These losses can occur due to process irreversibility, non-optimized heat exchange, or other non-ideal operating conditions. The graph of exergy losses of the equipment during the cryo-cooling process is shown in Fig. 9 c. Coolers 3 and 2 (Co-3 and Co-2) have the highest exergy losses, respectively. Total exergy loss has the largest share of input exergy. As mentioned, the input exergy is divided into three categories: useful exergy, exergy loss, and recovered work, it is shown in Fig. 9 d for the cryo-cooling process. Exergy losses for the equipment used in cycle 2 are shown in Fig. 9 e. The highest value is related to Cooler 3, which is equal to 272.1 kW. The exergy efficiency of different components is shown in Fig. 9 f. The results show that the highest yield is related to HX2. Also, the lowest value of exergy efficiency is related to the P-2. Exergy efficiency helps to identify where energy loss occurs in the system. By understanding exergy efficiency, liquefaction cycle design can change operational parameters and design aspects to improve overall system performance, leading to reduced energy consumption and cost savings. Higher exergy efficiency leads to more efficient use of energy resources, lower operating costs, and improved economic performance of the hydrogen liquefaction process. The power of the equipment used in cycle 2 is given in Table 12. The highest power is related to compressors. Among the compressors, C-3 (1333.45 kW) has the largest amount of power. Among the equipment, pumps have the lowest amount of power.

**Table 11:** Cycle 2 stream information.

Stream ID	Temperature T (°C)	Pressure p (kPa)	Mass flow $\dot{m}$ (kg/h)	Para (%)	Mass enthalpy h (kJ/kg)	Mass entropy s (kJ/kg-°C)	Mass exergy e (kJ/kg)
${\text{Feed}\;H}_{2}$	25	2100	500	25	5.84	54.29	3735.79
H1	−90.2	2100	500	25	−1654.77	47.24	4175.58
H2	−156.04	2100	500	25	−2620.7	40.68	5163.95
H3	−154.96	2100	500	33.59	−2620.71	42.24	5150.49
H4	−200.42	2100	500	33.59	−3323.71	34.7	6695.29
H5	−197.41	2100	500	51.89	−3323.46	37.77	6602.12
H6	−227	2100	500	51.89	−3854.48	28.74	8761.12
H7	−223.13	2100	500	77.61	−3854.53	32.67	8456.72
H8	−241.99	2100	500	77.61	−4433.86	17.67	12348.56
H9	−239.94	2100	500	95.68	−4433.92	20.1	11934.97
H10	−252.5	2100	500	95.68	−4731.24	8.96	14957.15
$LH_{2}$	−252.16	130	500	95.68	−4731.24	10.04	14637.23
LNG	−160	120	2170		−5167.14	4.29	957.9
L1	−156.5	3000	2170		−5158.72	4.31	959.01
L2	−96	3000	2170		−4936.15	5.84	725.49
L3	−96	3000	237		−4936.15	5.84	725.49
L4	−96	3000	1933		−4936.15	5.84	725.49
L5	−70.35	3000	1933		−4606.59	7.61	527.25
L6	10	3000	1933		−4358.97	8.66	461.05
L7	10	3000	237		−4358.97	8.66	461.05
R1	−68.87	160	1480		−184.79	2.88	90.08
R2	−95	160	1480		−615.22	0.6	340.48
R3	−93.46	2480	1480		−609.89	0.61	343.1
R4	−31.39	2480	1480		−465.02	1.3	282.97
R5	10.01	2480	1480		−67.12	2.78	238.21
1	23	110	4166		−10.47	20.8	50.91
2	186.77	275	4166		840.46	21.19	787.39
3	25	275	4166		−0.19	18.94	618.32
4	24.49	275	5605		−2.86	18.93	618.32
5	188.96	687.1	5605		851.79	19.31	1358.57
6	25	687.1	5605		−0.48	17.03	1185.44
7	189.83	1717.1	5605		856.13	17.42	1927.6
8	25	1717.1	5605		−1.13	15.13	1752.78
9	25	1717.1	1902		−1.13	15.13	1752.78
10	−163.33	1717.1	1902		−982.84	9.92	2324.16
11	−202.96	434.6	1902		−1186.24	10.46	1960.32
12	−228.25	110	1902		−1316.52	11	1669.5
13	−201.42	110	1902		−1176.91	13.43	1082.1
14	−165.9	110	1902		−992.18	15.53	643.08
15	23	110	1902		−10.47	20.81	50.91
16	25	1717.1	3703		−1.13	15.13	1752.78
17	−224.8	1717.1	3703		−1307.26	5.58	3293.08
18	−246.51	275	3703		−1413.49	6.34	2961.58
19	−246.51	275	1439		−1413.49	6.34	2961.58
20	−228.2	275	1439		−1317.32	9.09	2237.9
21	23	275	1439		−10.59	18.9	618.35
22	−246.51	275	2264		−1413.49	6.34	2961.58
23	−253.54	110	2264		−1448.69	6.66	2828.92
24	−241	110	2264		−1383.03	9.25	2122.37
25	−228.16	110	2264		−1316.22	11	1667.79
26	23	110	2264		−10.47	20.81	50.91

**Table 12:** Equipment power cycle 2.

Equipment		Power (kW)
Comps	C-1	984.71
	C-2	1330.94
	C-3	1333.45
Exps	E−1	48.38
	E−2	107.46
	E−3	68.83
	E−4	109.27
	E−5	22.14
Pumps	P-1	2.19
	P-2	5.08

### 5.3 Results and discussion of the proposed cycle 3

**Fig. 10:**
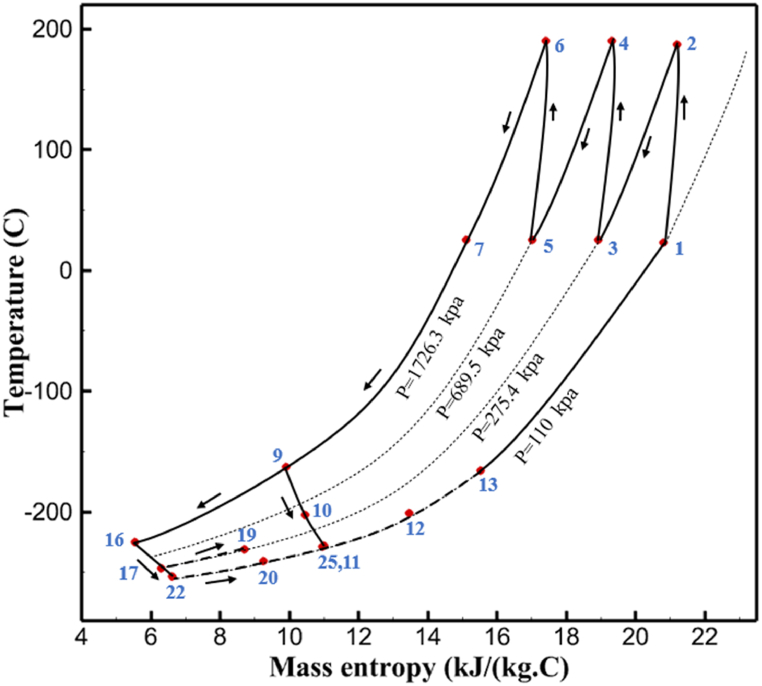
T-s diagram cycle 3.

**Fig. 11:**
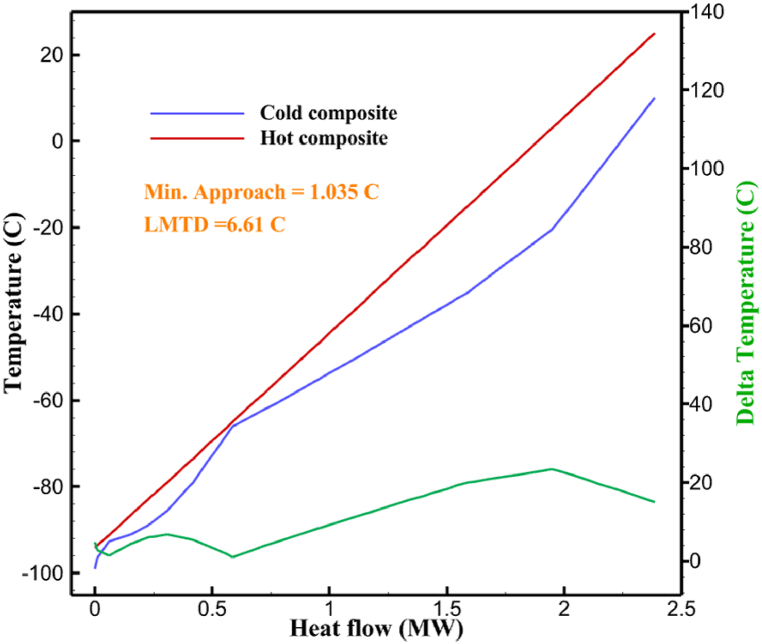

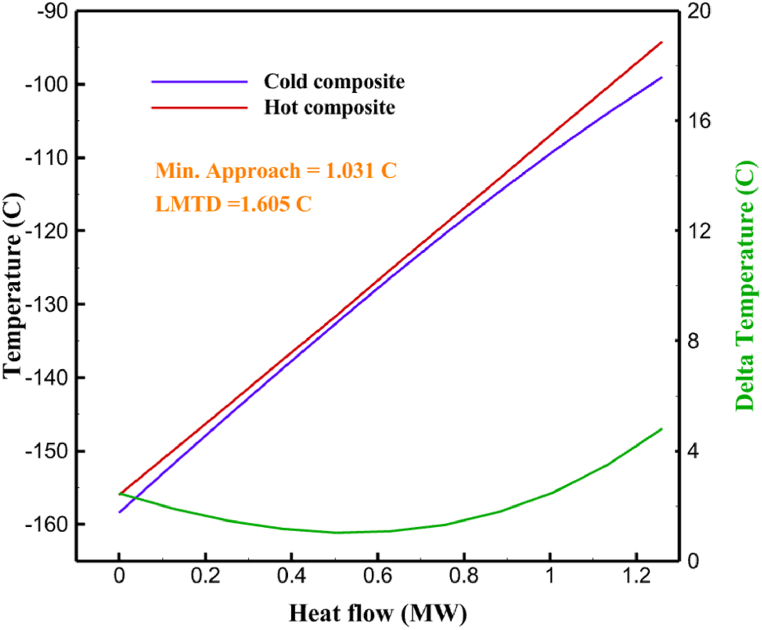

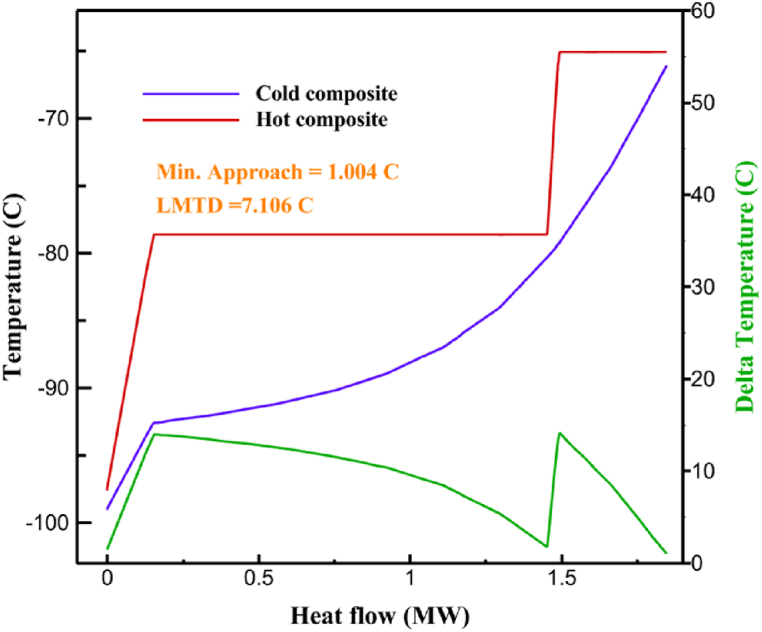

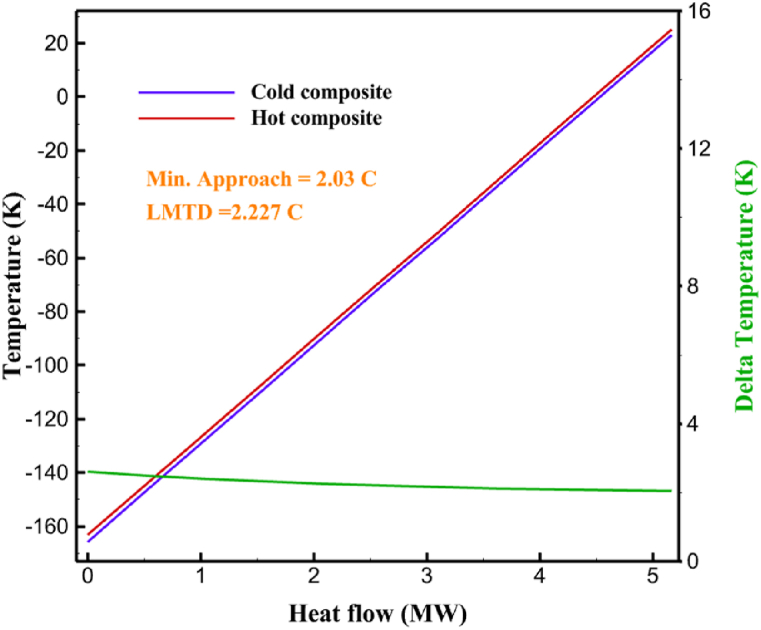

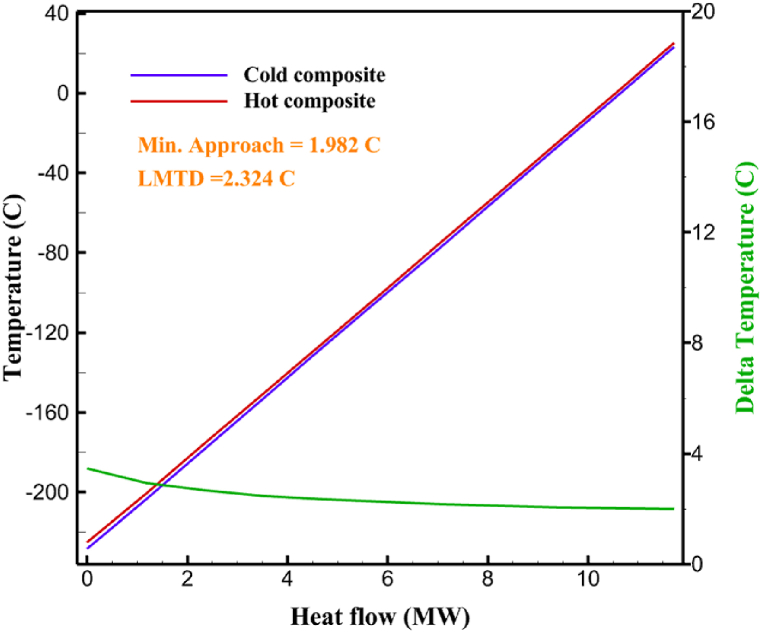

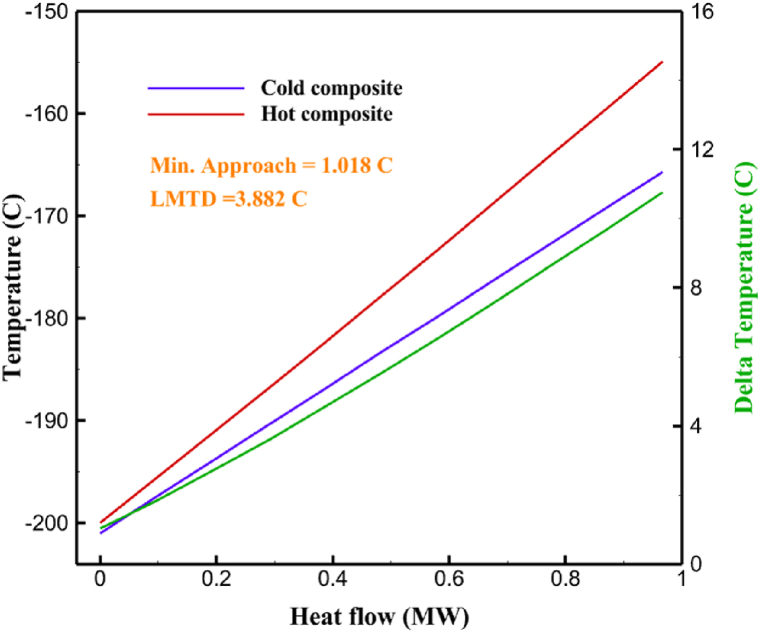

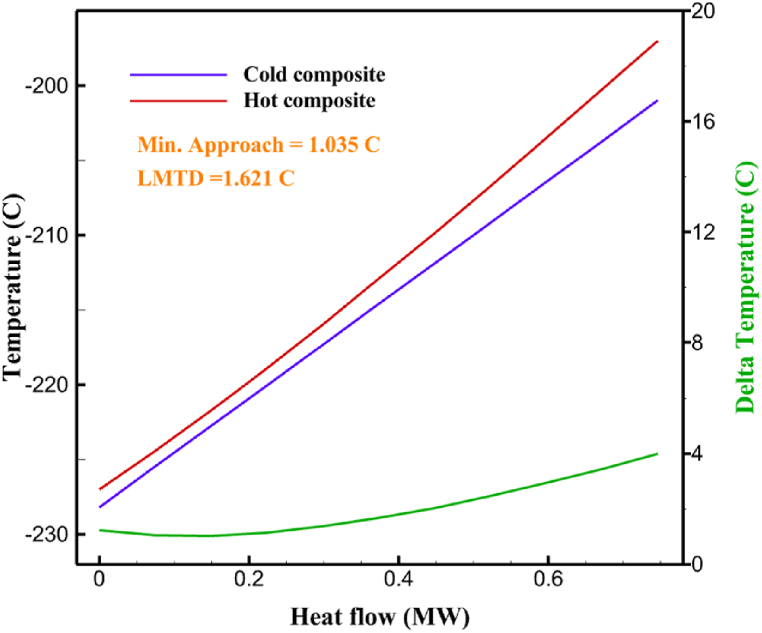

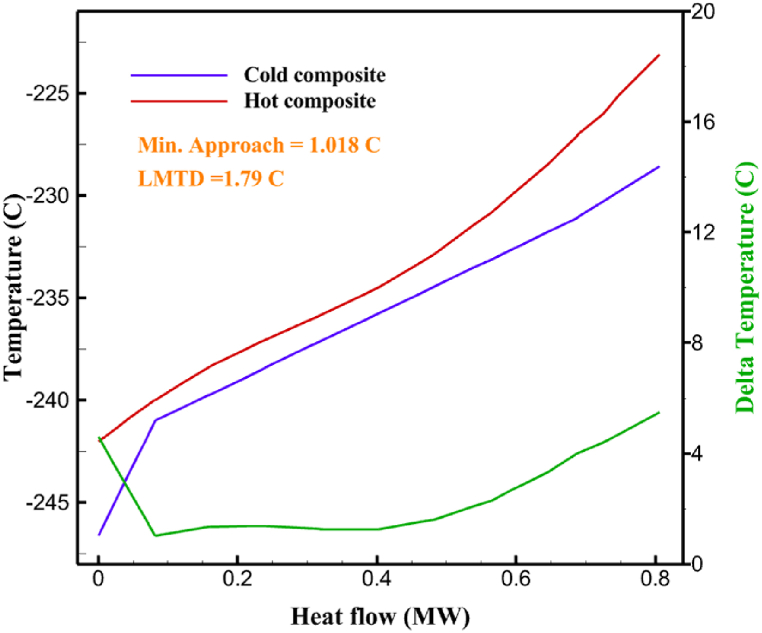

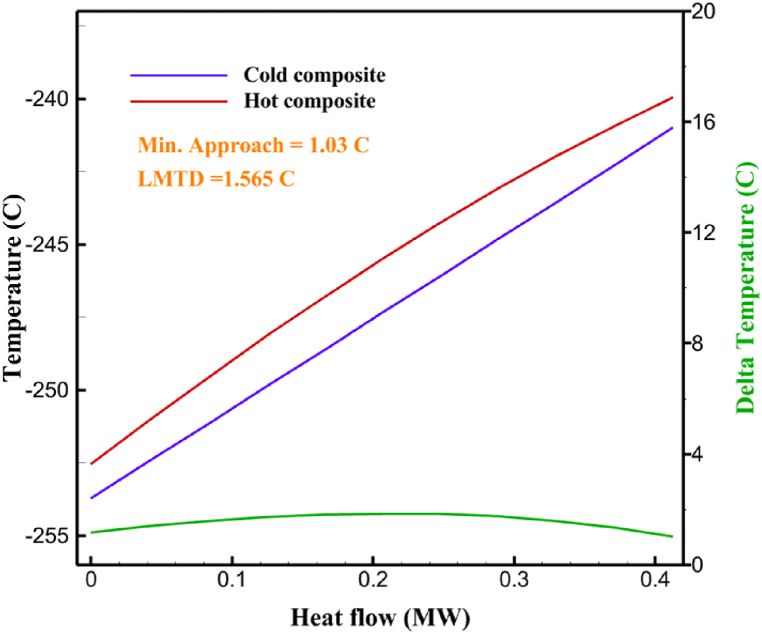
a HX1 information cycle 3. Fig. 11b HX2 information cycle 3. Fig. 11c HX3 information cycle 3. Fig. 11d HX4 information cycle 3. Fig. 11e HX5 information cycle 3. Fig. 11f HX6 information cycle 3. Fig. 11g HX7 information cycle 3. Fig. 11h HX8 information cycle 3. Fig. 11i HX9 information cycle 3.

**Fig. 12:**
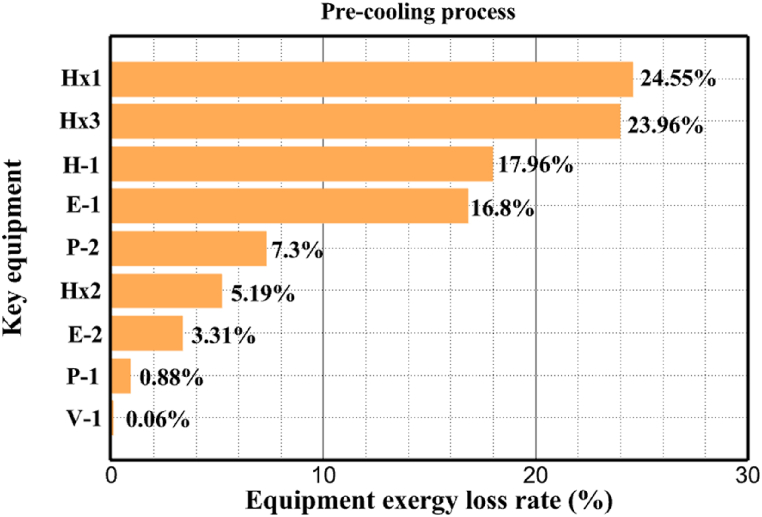

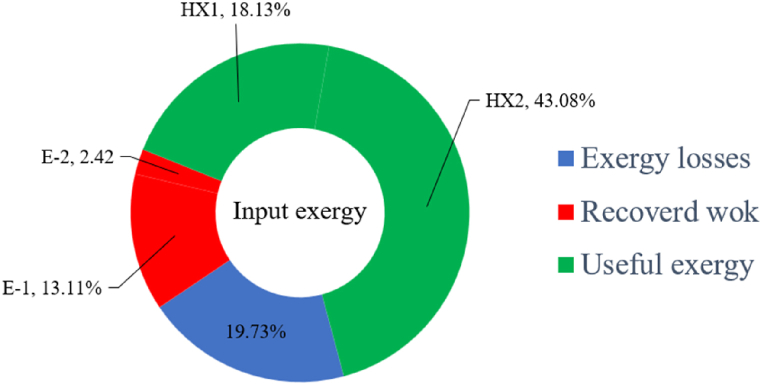

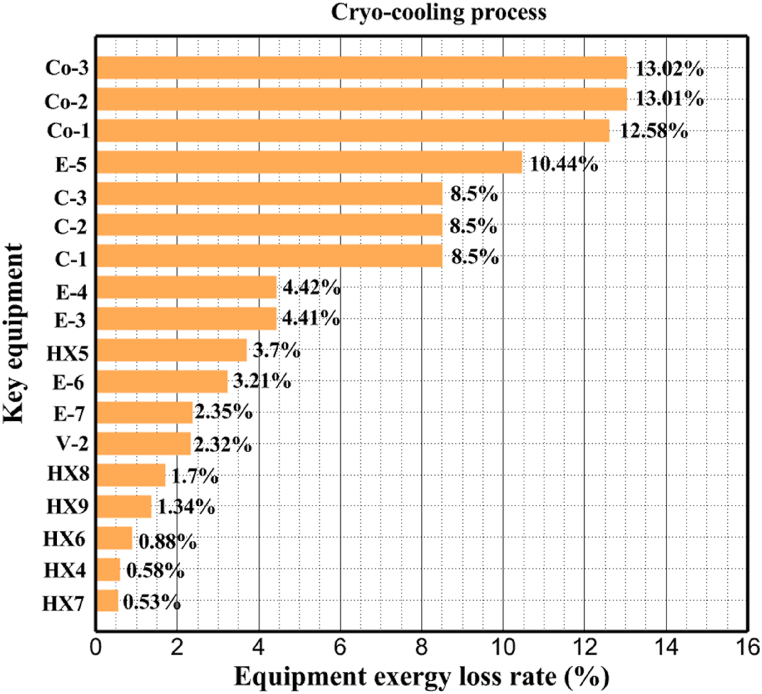

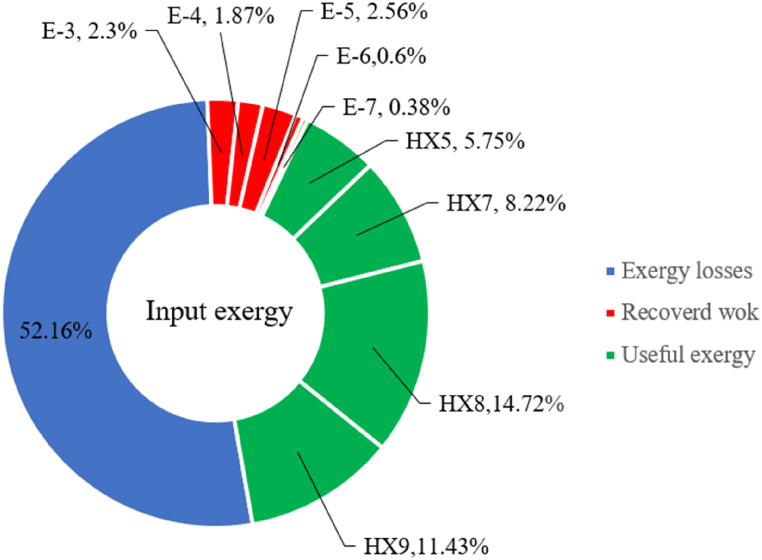

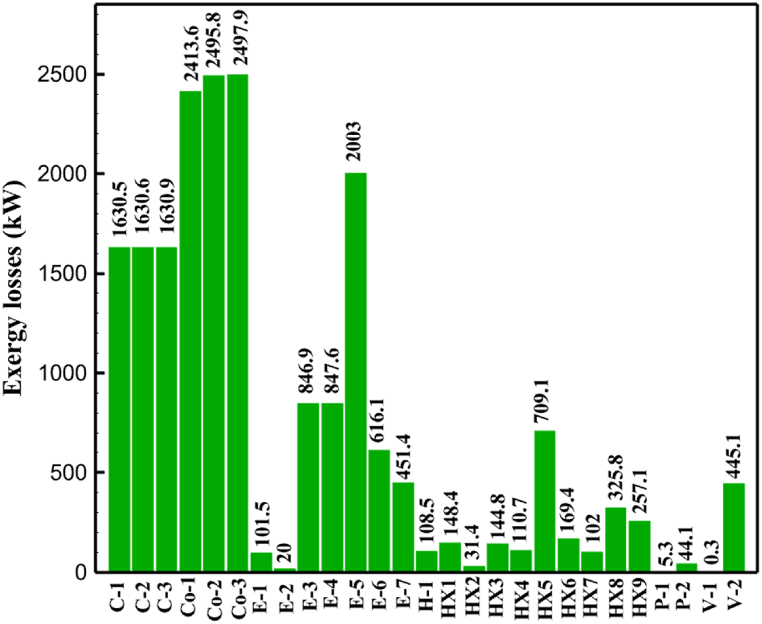

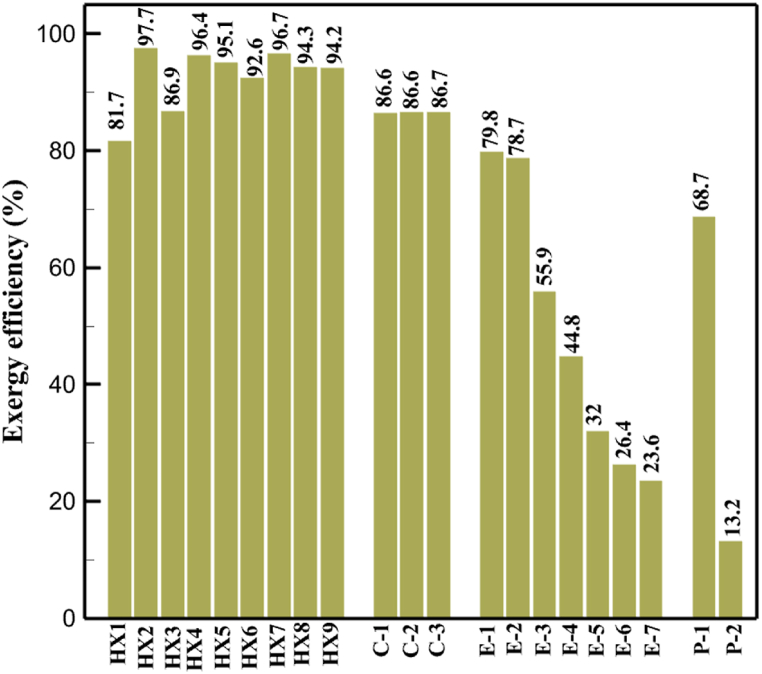
a Equipment exergy loss rate in pre-cooling process cycle 3. Fig. 12b Use of input exergy in pre-cooling process cycle 3. Fig. 12c Equipment exergy loss rate in cryo-cooling process cycle 3. Fig. 12d Use of input exergy in pre-cooling process cycle 3. Fig. 12e Exergy losses each equipment cycle 3. Fog 12.f Exergy efficiency of equipment cycle 3.

Table 13 provides information on the streams of the third cycle. This table covers all of the information required for a thermodynamic study of the cycle and equipment. Also, the percentage of hydrogen para is also displayed in the table, which shows that the cycle fulfills the necessary obligations regarding the percentage of hydrogen para. Fig. 10 depicts the temperature-entropy diagram during the cryo-cooling cycle 3. Like the previous two cycles, includes four constant pressure lines. It is worth noting that in the freezing cooling portion, the incoming and exiting streams from the mixer and separators have identical entropy. The connection between temperature and heat flow for both cold and hot flows, LMTD, min approach and delta temperature between these flows, are shown in the diagram in Fig. 11a to 11 i for each heat exchanger in cycle 3. Helium self-cooling heat exchangers (HX4 and HX5) in the cycle have a min approach approximately 2 °C, which is one of the important requirements to increase the efficiency of cryo-cooling. Fig. 12 a shows the exergy loss rate of different equipment parts in the pre-cooling process. HX1 has the highest exergy loss rate (23.96 %), which is due to inefficiency in heat transfer. In general, high exergy losses occurred in heat exchangers (HX1 and HX3), heaters/evaporators (H-1) and expander (E−1). Fig. 12 b shows the distribution of input exergy between exergy losses, recycled work and useful exergy for pre-cooling. HX2 has the highest useful exergy ratio, which shows that it transfers heat efficiently. This can be due to optimal operating conditions. Exergy loss rates in different equipment for cryo-cooling are shown in Fig. 12 c. Coolers (Co-1, Co-2, Co-3) have the highest exergy loss rates (13.02 %, 13.01 % and 12.58 %). Among the heat exchangers (HX5, HX6, HX7, HX8, HX9), HX5 and HX8 show a higher exergy loss of 3.7 % and 1.7 %, respectively. Losses can be due to temperature gradients or inefficiencies in heat transfer surfaces. Other heat exchangers such as HX6, HX7 and HX9 have lower losses, indicating better performance or lower load conditions. Among the expanders (E−3, E−4, E−5, E−6, E−7) E−5 shows a significant exergy loss rate of 10.44 %. The exergy loss rate of E−3 and E−4 is about 4.4 %, indicating moderate inefficiency. E−6 and E−7 show lower exergy losses, indicating better and more efficient operating conditions. All compressors have an exergy loss rate of 8.5 %. This could be due to high condensation temperatures leading to significant irreversibility. Fig. 12 d shows how the input exergy is used in the cryo-cooling section. As it is known, more than half of this input exergy is assigned to exergy losses. The exact amount of exergy loss in kW for all cycle equipment is given in Fig. 12 e. Fig. 12 f shows the exergy efficiency of the equipment. HX2 has the highest exergy efficiency. Exergy efficiency measures how effectively equipment can convert available energy into useful work. Higher exergy efficiency indicates better energy utilization, reduced waste, and improved overall process efficiency. Operating costs can be significantly reduced by identifying and optimizing equipment with low exergy efficiency. Efficient equipment requires less input energy for the same output, which reduces energy consumption and associated costs. Overall, focusing on exergy efficiency helps improve energy use, reduce costs, increase system performance, and support continuous optimization in hydrogen liquefaction processes. Table 14 shows the power of cycle components.

**Table 13:** Cycle 3 stream information.

Stream ID	Temperature T (°C)	Pressure p (kPa)	Mass flow $\dot{m}$ (TPD)	Para (%)	Mass enthalpy h (kJ/kg)	Mass entropy s (kJ/kg-°C)	Mass exergy e (kJ/kg)
${\text{Feed}\;H}_{2}$	25	2100	120	25	5.84	54.29	3735.79
H1	−94.22	2100	120	25	−1713.11	46.92	4213.41
H2	−156.02	2100	120	25	−2620.33	40.69	5163.39
H3	−154.93	2100	120	33.62	−2620.35	42.24	5149.88
H4	−199.98	2100	120	33.62	−3316.35	34.8	6673.28
H5	−197.02	2100	120	51.61	−3316.39	37.82	6582.7
H6	−227	2100	120	51.61	−3853.7	28.71	8760.42
H7	−223.09	2100	120	77.62	−3853.76	32.68	8452.82
H8	−242.01	2100	120	77.62	−4434.23	17.66	12351.6
H9	−239.97	2100	120	95.52	−4434.28	20.08	11941.1
H10	−252.54	2100	120	95.52	−4731.42	8.93	14967.1
$LH_{2}$	−252.16	130	120	95.52	−4731.42	10	14646.6
LNG	−160	120	520.8		−5167.14	4.29	957.9
L1	−158.46	3000	520.8		−5158.72	4.31	959.01
L2	−99.01	3000	520.8		−4949.68	5.76	734.92
L3	−99.01	3000	80.9		−4949.68	5.76	734.92
L4	−99.01	3000	439.9		−4949.68	5.76	734.92
L5	−6610	3000	439.9		−4586.93	7.71	518.29
L6	10	3000	439.9		−4358.97	8.67	461.05
L7	10	3000	80.9		−4358.97	8.67	461.05
R1	−96.5	2831.9	364.9		−7504.08	−0.25	315.14
R2	−20.5	2831.9	364.9		−7344.65	0.5	252.67
R3	10	2831.9	364.9		−6958.29	1.88	226.97
R4	−65.1	219.2	364.9		−7053.35	1.96	107.87
R5	−65.1	219.2	62.52		−7445.11	0.01	277.52
R6	−65.1	219.2	64.98		−6972.35	2.35	72.79
R7	−65.1	219.2	127.5		−7204.16	1.23	173.18
R8	−97.5	−127.5	127.5		−7508.06	−0.25	312.5
R9	−97.45	110.4	127.5		−7508.06	−0.25	312.27
R10	−65.1	219.2	302.4		−6972.35	2.35	72.79
R11	−65.1	219.2	237.4		−6972.35	2.35	72.79
R12	−78.6	110.4	237.4		−6999.31	2.37	38.54
R13	−97.57	110.4	237.4		−7508.27	−0.25	312.42
R14	−97.53	110.4	364.9		−7508.2	−0.25	312.37
1	23	110	1229.3		−10.47	20.81	50.91
2	187.08	275.4	1229.3		842.07	21.19	788.85
3	25	275.4	1229.3		−0.2	18.93	619.22
4	190.2	689.5	1229.3		858.2	19.32	1363.01
5	25	689.2	1229.3		−0.48	17.03	1187.6
6	190.23	1726.3	1229.3		858.21	17.41	1931.65
7	25	1726.3	1229.3		−1.14	15.12	1756.09
8	25	1726.3	455		−1.14	15.12	1756.09
9	−163.1	1726.3	455		−981.66	9.92	2325.39
10	−202.87	435.8	455		−1185.76	10.46	1960.47
11	−228.23	110	455		−1316.41	11	1668.88
12	−201	110	455		−1174.7	13.47	1075.17
13	−165.7	110	455		−991.14	15.54	641.23
14	22.97	110	455		−10.63	20.8	50.91
15	25	1726.3	774.3		−1.14	15.12	1756.09
16	−225.1	1726.3	774.3		−1308.92	5.54	3304.85
17	−246.6	279.3	774.3		−1414.01	6.29	2976.25
18	−246.6	279.3	238.4		−1414.01	6.29	2976.25
19	−231.1	279.3	238.4		−1332.52	8.71	2336.47
20	−241	110	238.4		−1383.03	9.25	2122.37
21	−246.6	279.3	535.9		−1414.01	6.29	2976.25
22	−253.71	110	535.9		−1449.56	6.62	2841.37
23	−241	110	535.9		−1383.03	9.25	2122.37
24	−241	110	774.3		−1383.03	9.25	2122.37
25	−228.56	110	774.3		−1318.16	10.96	1678.79
26	23.02	110	774.3		−10.38	20.81	50.91

**Table 14:** Equipment power cycle 3.

Equipment		Power (kW)
Comps	C-1	12130
	C-2	12213.3
	C-3	12217.4
Exps	E−1	401.48
	E−2	74.08
	E−3	1074.87
	E−4	688.02
	E−5	941.83
	E−6	220.5
	E−7	139.36
Pumps	P-1	17.04
	P-2	50.79

### 5.4 UA heat exchanger, SEC, COP and EXE

**Fig. 13:**
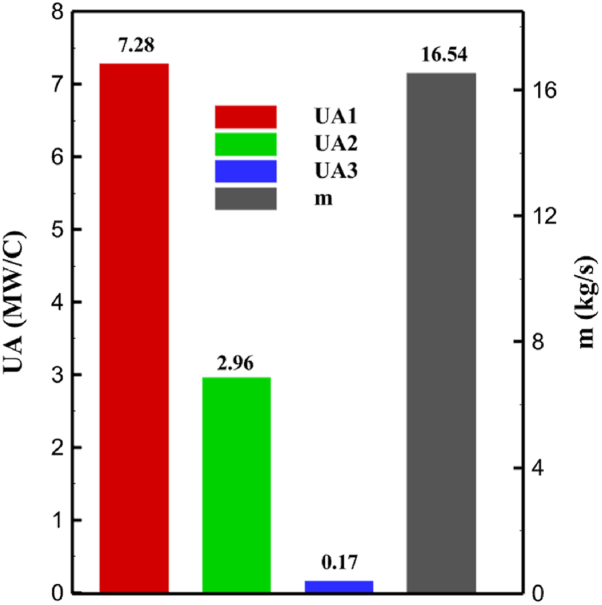

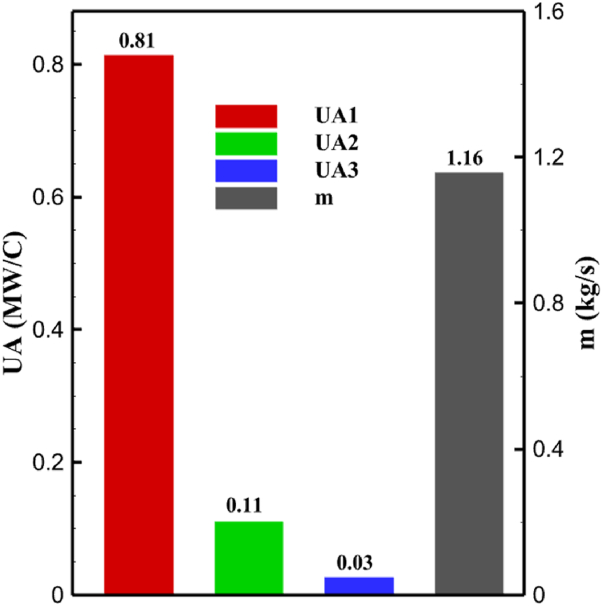

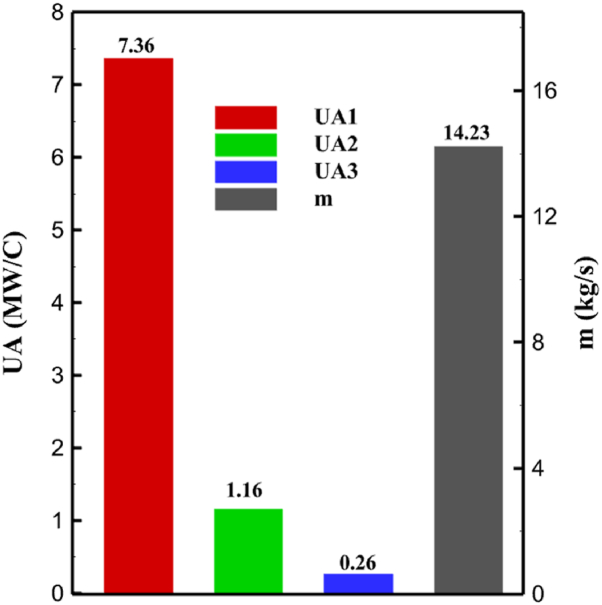

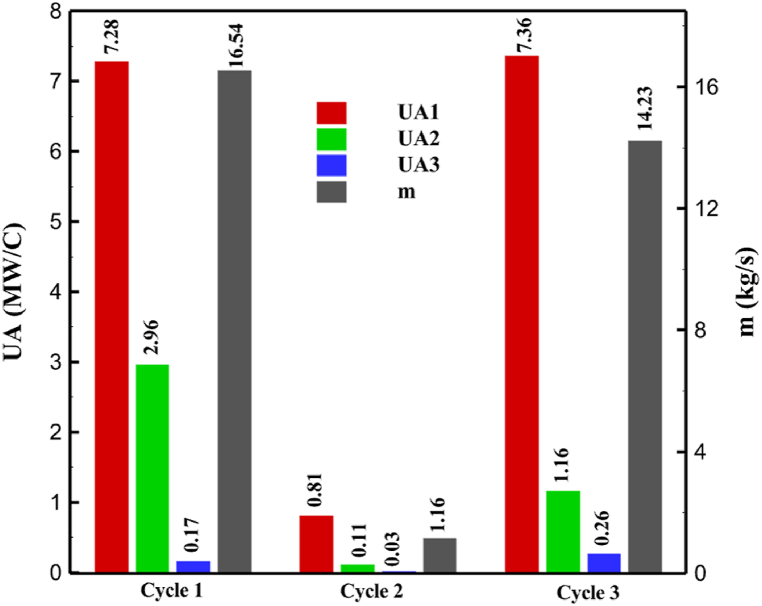
a UA value for cycle 1 heat exchangers. Fig. 13b UA value for cycle 2 heat exchangers. Fig. 13c UA value for cycle 3 heat exchangers. Fig. 13d Comparison of UA in 3 cycles.

**Fig. 14:**
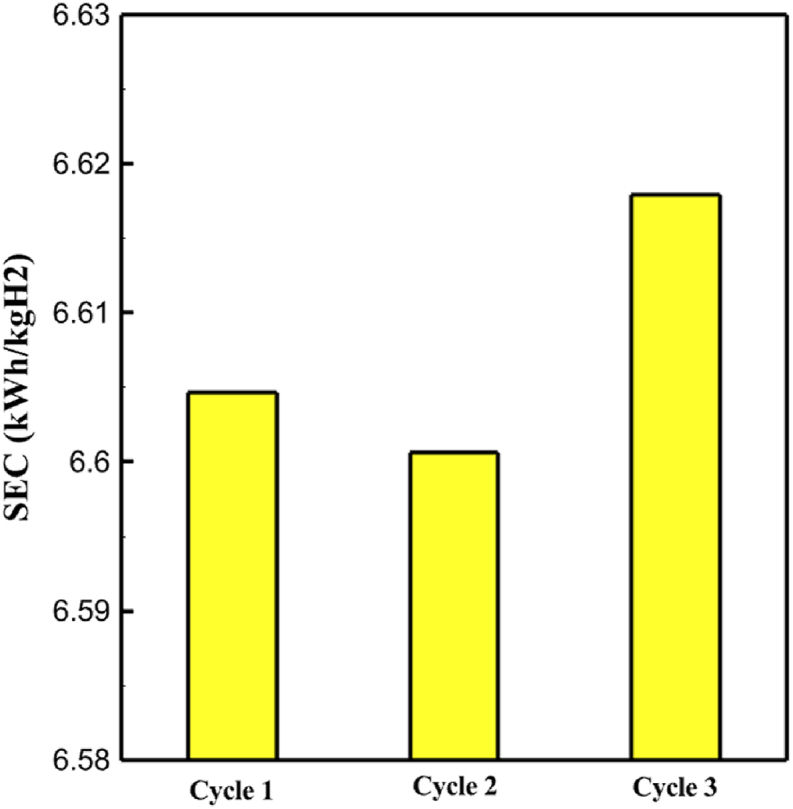

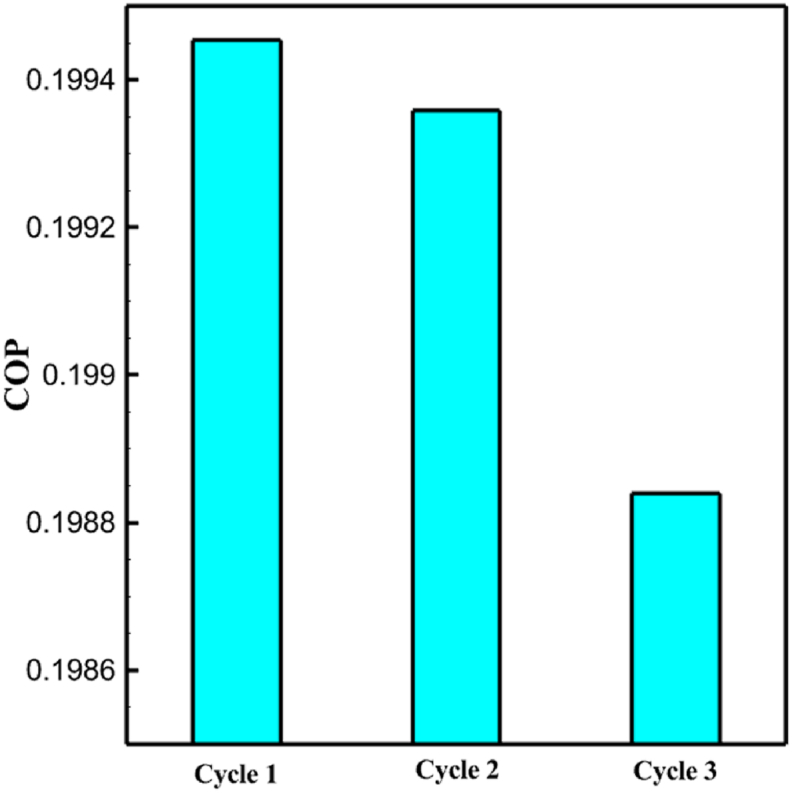

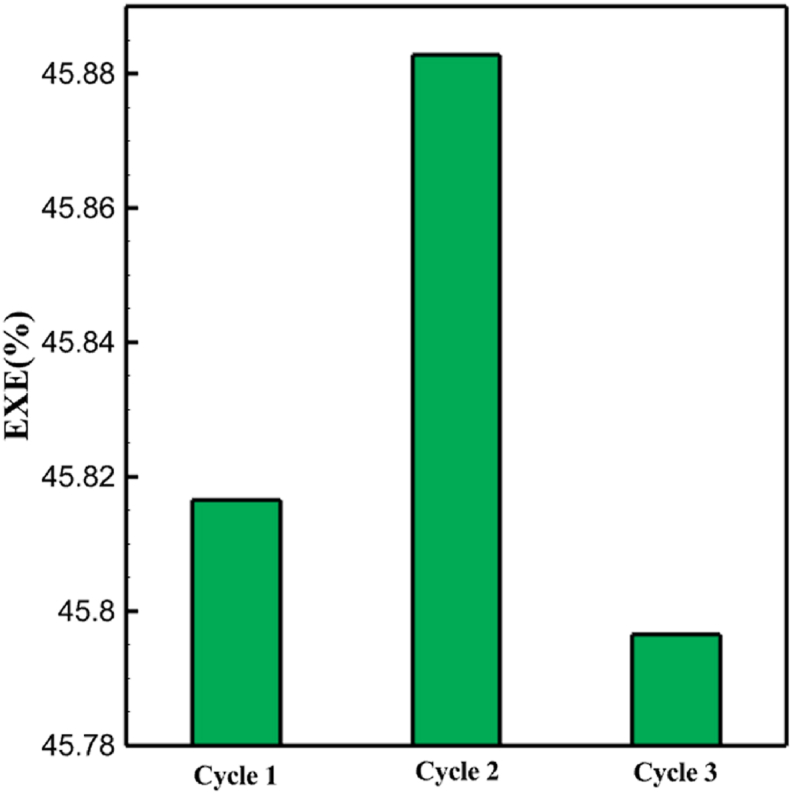
a Comparison of SEC 3 cycles. Fig. 14b Comparison of COP 3 cycles. Fig. 14c Comparison of EXE 3 cycles.

One might consider the equipment purchase expenses of a hydrogen liquefaction plant to represent the capital costs. The equipment used in the cycle, such as heat exchangers, compressors, and expanders, is the main focus of capital cost analysis for liquefaction operations. The operating power is the primary factor influencing the compressor and expander's expenses. The power of the expanders and compressors for each cycle as mentioned earlier is listed in Table 10, Table 12, Table 14 respectively. The primary factor influencing heat exchanger costs is their size, which is determined by the total heat exchange surface. Calculating the coefficient of heat transfer in heat exchangers between hot and cold fluids is challenging, however. Consequently, one assessment index for heat exchanger expenses is the heat exchanger's UA value, which can be found by multiplying the heat exchange area by the heat transfer coefficient. The heat exchangers used in the cryo-cooling process of the cycles are divided into three groups: helium self-cooling, hydrogen gas cooling and liquid hydrogen cooling, in order to more accurately compared the costs of cryo-cooling process heat exchangers in 3 cycles. For example, in cycle 1, HX6 is self-cooling helium heat exchanger. Also, HX2, HX3 and HX5 is hydrogen gas cooling heat exchanger and HX5 is liquid hydrogen cooling heat exchanger. UA1, UA2, and UA3 denote the relevant UA values for the three heat exchanger classifications. Fig. 13a to 13 c shows this value for the three investigated cycles. Also, the right axis of the diagrams shows the helium mass flow rate used. For example, for the cycle 3, the value of UA1 is equal to 7.36 MW/°C. This UA includes HX 4 and HX5. The value of UA 2 is equal to 1.16 MW/°C. This UA includes HX6, HX7, and HX8. UA value is 3 times 0.26 MW/°C. This UA includes HX9. Also, Fig. 13 d simultaneously shows the value of UA for the cycles along with the mass flow rate of helium in a graph. The calculated values of SEC, COP, and EXE for three cycles are presented graphically in Fig. 14a to 14 c. A hydrogen liquefaction cycle's specific energy consumption (SEC) is an important measure of the process's energy efficiency. A lower SEC number suggests a more energy-efficient process, while a larger SEC value indicates increased energy consumption per kilogram of liquid hydrogen. A low SEC number indicates that the liquefaction process requires less energy to create the same quantity of liquid hydrogen, implying greater energy efficiency. The value of SEC for cycles 1, 2, and 3 is equal to 6.605 $\text{kWh}/{\text{kg}}_{LH_{2}}$ , 6.601 $\text{kWh}/{\text{kg}}_{LH_{2}}$ and 6.618 $\text{kWh}/{\text{kg}}_{LH_{2}}$, respectively (Fig. 14a). The coefficient of performance (COP) in a hydrogen liquefaction cycle is a useful metric of system efficiency. A higher COP number implies more efficiency, which means that the system uses less energy to accomplish the same amount of hydrogen liquefaction. COP values for the three examined cycles are equal to 0.19945, 0.19936 and 0.19884, respectively. This topic is also shown in Fig. 14 b. The value of exergy efficiency (EXE) in a hydrogen liquefaction cycle is an important measure that shows how well the process converts available energy into useful work. Typically, the EXE value is expressed as a percentage, with higher values indicating more efficient processes. The values of 45.816 %, 45.883 % and 45.797 % correspond to EXE cycles 1, 2, and 3, respectively, which are also shown in Fig. 14 c.

### 5.5 Limitations

The simulation of hydrogen liquefaction cycles in Aspen HYSYS faces several limitations, primarily stemming from the accuracy of thermodynamic property predictions at the extremely low temperatures involved. Common equations of state Peng-Robinson struggle to accurately capture the behavior of hydrogen and mixed refrigerants at cryogenic conditions, affecting enthalpy calculations, phase equilibrium predictions, and ortho-para conversion estimations. This can lead to deviations in process parameters like energy consumption, cooling requirements, and overall process efficiency. Additionally, accurately modeling complex phenomena such as heat exchanger performance, pressure drop in piping and equipment, and ortho-para conversion kinetics can be challenging, further contributing to discrepancies between simulation results and real-world plant operation.

## 6 Conclusion

In this study, the thermodynamic analysis of three hydrogen liquefaction cycles was performed. The first cycle is the hydrogen liquefaction process, which uses a double-pressure J-B cascade cycle for hydrogen cooling. In the refrigeration cycles, LNG and helium are utilized as refrigerants for hydrogen cryo-cooling. With the aid of an organic Rankine cycle—which has been studied as a hydrogen pre-cooling cycle in cycle 2—the processes of hydrogen liquefaction and gasification of liquid natural gas are merged. In the hydrogen liquefaction sector, it should be mentioned that a combination of hydrogen and liquefied natural gas pre-cooling technologies shows promise. For hydrogen cooling, a refrigeration system with two double-pressured J-B cascade cycles also taken into consideration. Improved J-B cascade refrigeration cycles and LNG regasification process support the DORC in cycle 3. Some of the results of this research are given below.

• In all three liquefaction cycles, the third cooler (the cooler after the last compressor) has the most exergy losses.
• In all three examined cycles, more than 50 % of the input exergy is consumed in the form of exergy losses in the cryo-cooling stage.
• In cycle 1, the highest exergy efficiency is related to HX3 and the lowest exergy efficiency is related to P-1. In cycle 2 and 3, however, the highest exergy efficiency is related to HX2 and the lowest is related to LNG pump.
• In the thermodynamic analysis of all three cycles, it was found that the compressors have the most power. Among the compressors, C-3 has the highest amount of power in all three cycles.
• The value of SEC for cycle 1,2, and 3 is equal to 6.605 $\text{kWh}/{\text{kg}}_{LH_{2}}$ , 6.601 $\text{kWh}/{\text{kg}}_{LH_{2}}$ and 6.618 $\text{kWh}/{\text{kg}}_{LH_{2}}$, respectively.
• COP values for the three examined cycles are equal to 0.19945, 0.19936 and 0.19884, respectively.
• The values of 45.816 %, 45.883 % and 45.797 % correspond to EXE cycles 1, 2, and 3, respectively,
• In all three cycles, helium self-cooling heat exchangers have a min approach temperature equal 2 °C. Other heat exchangers in cycles have a Min approach of less than 2 °C. These minimum temperatures are considered due to the operating conditions and better efficiency of the cycles, and the results indicate a good agreement between the hot and cold composite charts.

Finally, it should be noted that hydrogen is a clean fuel that has gained a lot of attention as an alternative to conventional fuels since it is the most plentiful element in the world and burns cleanly. Thermodynamic study of liquefaction cycles on various sizes and with various pre-cooling and cryo-cooling cycles allows scientists to develop an ideal liquefaction plant based on these results. Scientists can discover new proposed cycles by examining the obtained results and energy and exergy analyzes and other analyses.

## 7 Future direction

Future research on energy and exergy analysis of hydrogen liquefaction cycles should focus on optimizing process efficiency and reducing energy consumption. This includes exploring advanced cycle configurations, such as the integration of renewable energy sources and the development of novel refrigeration technologies. Additionally, further investigation into the exergy destruction points within the cycle can provide insights into minimizing irreversibilities, thereby improving overall performance. Accurate simulations of hydrogen liquefaction cycles are pivotal in shaping the future of hydrogen research and its broader energy implications. Precise simulations enable researchers to optimize existing processes, identify potential bottlenecks, and propose innovative designs for more efficient and cost-effective liquefaction cycle. This can lead to the development of more compact and accessible hydrogen liquefaction infrastructure, accelerating its widespread adoption. The ability to simulate hydrogen liquefaction under various conditions allows researchers to study the impact of different feed streams, refrigerant mixtures, and operating parameters on the overall process efficiency.

## CRediT authorship contribution statement

**Mehdi Mahboobtosi:** Validation, Software, Methodology, Data curation, Writing – original draft, Writing – review & editing. **D. D. Ganji:** Writing – review & editing, Formal analysis. **Mofid Gorji:** Supervision, Conceptualization. **Khashayar Hosseinzadeh:** Investigation, Supervision.

## Declaration of competing interest

The authors declare that they have no known competing financial interests or personal relationships that could have appeared to influence the work reported in this paper.
